# Loss of FOXA2 induces ER stress and hepatic steatosis and alters developmental gene expression in human iPSC-derived hepatocytes

**DOI:** 10.1038/s41419-022-05158-0

**Published:** 2022-08-16

**Authors:** Maryam Aghadi, Ramy Elgendy, Essam M. Abdelalim

**Affiliations:** 1 Diabetes Research Center, Qatar Biomedical Research Institute (QBRI), Hamad Bin Khalifa University (HBKU), Qatar Foundation (QF), Doha, Qatar; 2 College of Health and Life Sciences, Hamad Bin Khalifa University (HBKU), Qatar Foundation, Education City, Doha, Qatar; 3 Discovery Biology, Discovery Sciences, R&D, AstraZeneca, Gothenburg, Sweden

**Keywords:** Induced pluripotent stem cells, Stem-cell differentiation

## Abstract

FOXA2 has been known to play important roles in liver functions in rodents. However, its role in human hepatocytes is not fully understood. Recently, we generated FOXA2 mutant induced pluripotent stem cell (FOXA2^−/−^iPSC) lines and illustrated that loss of FOXA2 results in developmental defects in pancreatic islet cells. Here, we used FOXA2^−/−^iPSC lines to understand the role of FOXA2 on the development and function of human hepatocytes. Lack of FOXA2 resulted in significant alterations in the expression of key developmental and functional genes in hepatic progenitors (HP) and mature hepatocytes (MH) as well as an increase in the expression of ER stress markers. Functional assays demonstrated an increase in lipid accumulation, bile acid synthesis and glycerol production, while a decrease in glucose uptake, glycogen storage, and Albumin secretion. RNA-sequencing analysis further validated the findings by showing a significant increase in genes associated with lipid metabolism, bile acid secretion, and suggested the activation of hepatic stellate cells and hepatic fibrosis in MH lacking FOXA2. Overexpression of FOXA2 reversed the defective phenotypes and improved hepatocyte functionality in iPSC-derived hepatic cells lacking FOXA2. These results highlight a potential role of FOXA2 in regulating human hepatic development and function and provide a human hepatocyte model, which can be used to identify novel therapeutic targets for FOXA2-associated liver disorders.

## Introduction

The liver development and function are governed by an important set of transcription factors (TFs) such as HNF4A, TBX3, PROX1, FOXA1, FOXA2, FOXA3, HNF1α/β, HNF6 (ONECUT1), and C/EBPα [1]. These TFs control the expression of hepatic genes and work together to perform different functions. One of those key TFs is FOXA2 (HNF3; hepatocyte nuclear factor 3), which belongs to the family of forkhead class of TFs [2]. Studies carried out in animals have shown that mice lacking *Foxa2* die shortly after birth due to defects in notochord; therefore, Cre-Loxp system has been generated to retain the expression of *Foxa2* in axial mesoderm and it is later depleted in the foregut endoderm after day 8.5 [2]. This mouse model showed a normal induction and growth of hepatic developmental program due to the compensatory role played by its sister gene, *Foxa1* [2]. Double knockout for *Foxa1* and *Foxa2* in mid-gestation embryos revealed no embryo to surpass beyond day 10, indicating that both *Foxa1* and *Foxa2* are essential for hepatic specification [3]. From a developmental standpoint, previous studies have reported Foxa2 to hold paramount importance in decondensing chromatin in foregut endoderm required for the onset of hepatic transcriptional program [4]. Specifically, this decondensation is performed over the α-fetoprotein (AFP) gene promoter, which removes the chromatic block held over it and allows its transcription [4]. Similar function is performed at the maturation stage where binding of Foxa2 to nucleosomal site at the enhancer of Albumin (ALB) gene in active chromatin releases chromatin block held over its expression [5]. In addition, FOXA2 has been found to regulate functionality by regulating lipid metabolism and ketogenesis in liver during fasting [6]. Most of these studies have been carried out using animal models, limiting our knowledge on the regulatory role of FOXA2 in humans. A recent human study highlighted the role of FOXA2 in chromatin decondensation for gaining hepatic competency [7]; however, the exact role of FOXA2 in the development and function of human hepatocytes has not been well-studied.

The advent of induced pluripotent stem cell (iPSC) technology has enabled studying the role of various TFs by recapitulating the process of human development in-vitro. Our lab has recently made use of this technology to demonstrate FOXA2’s role in pancreatic beta cell development, and have found aberrant pancreatic development in the absence of FOXA2 by showing reduced expression of key TFs governing beta cell development [8]. In the current study, our aim was to use those FOXA2^−/−^ iPSC models to understand the role of FOXA2 during human hepatocyte development. Our findings showed that lack of FOXA2 led to defects in the development and function of iPSC-derived hepatocytes.

## Results

### Expression of FOXA2 during differentiation of iPSCs into hepatocytes

**Fig. 1:**
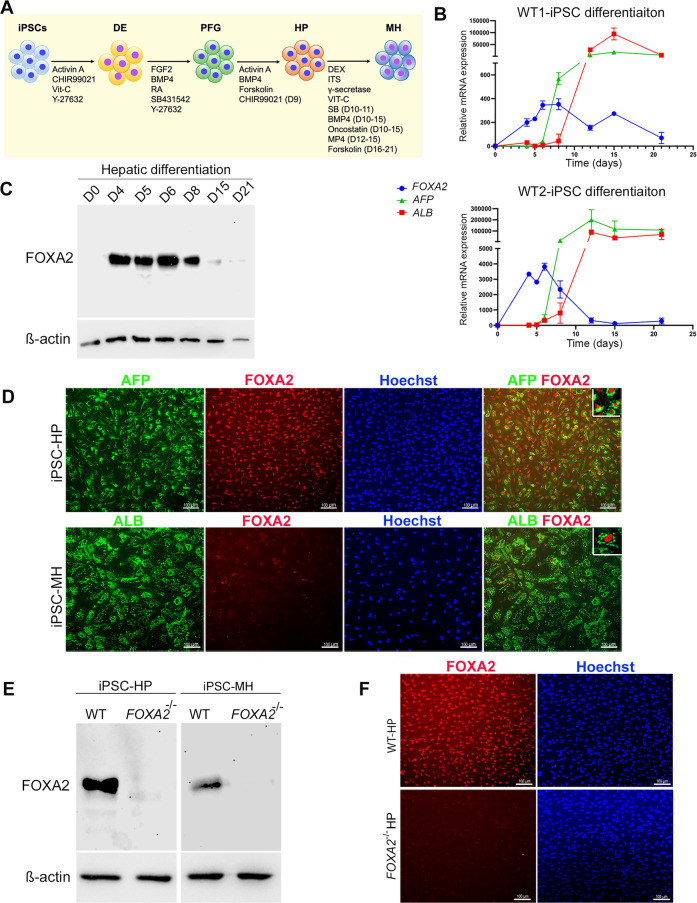
Expression of FOXA2 during differentiation of iPSCs into hepatocytes. **A** Schematic diagram of iPSC differentiation into hepatocytes. **B** Real-time PCR analysis for the mRNA expression of *FOXA2*, *AFP*, and *ALB* during hepatocyte differentiation. Data are presented as the fold change relative to day 0 of differentiation (*n* = 3). **C** Western blot analysis of FOXA2 expression levels during various stages of differentiation of iPSCs into hepatocytes. **D** Immunostaining of FOXA2 with the hepatic progenitor (HP) and mature hepatocyte (MH) markers, AFP and ALB, at the end of HP and MH stages, respectively. Western blotting (**E**) and immunofluorescence (**F**) analyses confirming the absence of FOXA2 protein in HP and MH derived from FOXA2^−/−^ iPSCs. DE definitive endoderm, PFG posterior foregut, HP hepatic progenitor, MH mature hepatocytes. The data are presented as mean ± SD. *p < 0.05, **p < 0.01, ***p < 0.001.

To determine the timeline of FOXA2 expression during hepatocyte development, iPSCs generated from two healthy controls (Ctr1 and Ctr2) were differentiated into all stages of hepatocyte development, including definitive endoderm (DE), posterior foregut (PF), HP and MH (Fig. 1A). Levels of FOXA2 during differentiation were examined using qRT-PCR (Fig. 1B) and Western blotting (Fig. 1C), which showed that the FOXA2 expression started during DE stage, and gradually increased its expression levels till reached its highest expression levels at days 6 and 8 (Fig. 1B, C). Following day 8, levels of FOXA2 start to decline, with little to no expression by the end of differentiation at the mature stage (Fig. 1B, C). Furthermore, immunostaining analysis confirmed these results, where the expression of FOXA2 was highly expressed and co-localized with AFP+ cells in HP at day 10 of differentiation. However, it was dramatically reduced in MH and only a few ALB+ cells co-expressed FOXA2 (Fig. 1D), indicating that the FOXA2 expression is required for early stages of hepatic development. A previous study reported that during fasting and starvation, FOXA2 expression is reactivated in liver cells [6]. To confirm if this is the same in iPSC-derived MH, we examined the expression of FOXA2 in MH under starved and normal culture conditions. Interestingly, we noticed a dramatic increase in the FOXA2 protein levels in iPSC-derived MH exposed to starvation (Supplementary Fig. 1). This indicates that FOXA2 can be reactivated in MH to protect the hepatocytes from the defects related to FOXA2 functions.

### Effect of FOXA2 deficiency on the development of iPSC-derived hepatocytes

In order to investigate the role of FOXA2 in the development and function of human hepatocytes, we established FOXA2 knockout iPSC models (FOXA2^−/−^iPSCs) using CRISPR/Cas9 as we recently reported [8]. We further confirmed the absence of FOXA2 in HP and MH derived from FOXA2^−/−^iPSCs using Western blotting and immunostaining (Fig. 1E, F). These FOXA2^−/−^ iPSCs along with their isogenic controls (WT) were used for subsequent experiments and the results were reproduced in the two different FOXA2^−/−^ iPSC lines. Morphologically, there was a clear difference between HP and MH. The HP cells were small, closely packed, and uniform in size; however, MH cells were larger, hexagonal in shape with different sizes, indicating a hepatocyte-like morphology (Supplementary Fig. 1). There was no clear difference between WT-HP and FOXA2^−/−^ HP; however, there was a clear difference between WT-MH and FOXA2^−/−^ MH. The FOXA2^−/−^ MH cells were bigger in size than WT-MH and contained vacuolated structures (Supplementary Fig. 2), which may indicate intracellular lipid accumulation.

**Fig. 2:**
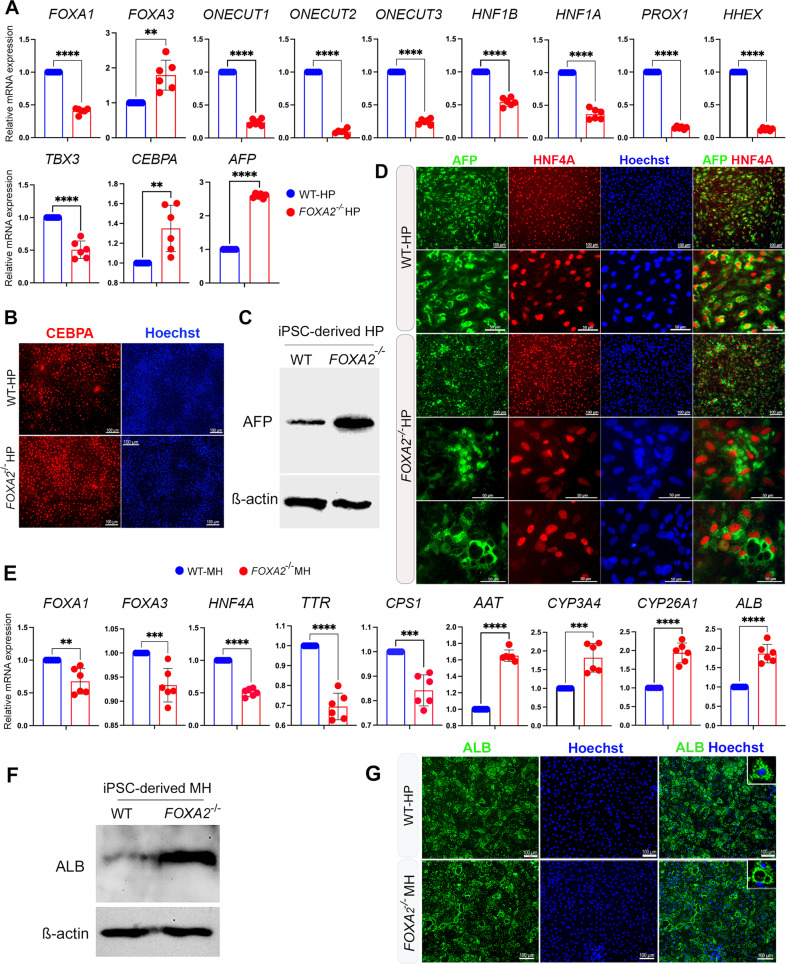
Effect of FOXA2 knockout on iPSC-derived hepatocytes. **A** RT-qPCR analysis showing the mRNA expression of hepatic progenitor (HP) markers*, FOXA1*, *FOXA3*, *ONECUT1*, *ONECUT2*, *ONECUT3*, *HNF1B*, *HNF1A*, *PROX1*, *HHEX*, *TBX3, CEBPA*, and *AFP* in FOXA2^−/−^ HP relative to wild type (WT) controls (*n* = 6). **B** Immunofluorescence images showing the expression of CEBPA in HP derived from WT-iPSCs and FOXA2^−/−^iPSCs. **C** Western blot analysis showing the upregulation of AFP in HP lacking FOXA2 compared to WT controls. **D** Immunofluorescence analysis showing co-expression of AFP and HNF4A in HP derived from WT-iPSCs and FOXA2^−/−^iPSCs. Note the pattern of AFP expression in FOXA2^−/−^HP. **E** RT-qPCR analysis showing the mRNA expression of mature hepatocyte (MH) markers*, FOXA1*, *FOXA3*, *HNF4A*, *AAT*, *TTR*, *CPS1, CYP3A4*, *CYP26A1*, and *ALB* in *FOXA2*^*−/−*^ MH relative to WT controls (*n* = 6). **F** Western blotting showing the upregulation of ALB protein in FOXA2^−/−^ MH compared to WT. **G** Immunofluorescence showing ALB protein expression in FOXA2^−/−^MH compared to WT controls. Note the ALB distribution around cytoplasmic vacuoles. Nuclei were counterstained with Hoechst. The data are presented as mean ± SD. *p < 0.05, **p < 0.01, ***p < 0.001.

The effect of FOXA2 loss on HP development was assessed by quantifying the expression of key developmental markers using qRT-PCR at day 10 of differentiation. The results showed a significant reduction in the mRNA levels of *FOXA1*, *ONECUT1*, *ONECUT2*, *ONECUT3*, *HNF1B*, *HNF1A*, *PROX1*, *HHEX*, and *TBX3*, while *FOXA3, CEBPA*, and *AFP* levels were significantly upregulated in the absence of FOXA2 (Fig. 2A, Supplementary Fig. 3). The increase in CEBPA and AFP was further validated by immunostaining and Western blotting, respectively, which showed their relative increase in HP lacking FOXA2 compared to WT controls (Fig. 2B, C). Interestingly, although immunostaining analysis did not show a remarkable alteration in the expression levels of AFP between WT-HP and FOXA2^−/−^ HP, we noticed an aberrant distribution pattern of AFP (Fig. 2D). AFP being a cytoplasmic protein showed a homogenous intracellular distribution in WT-HP, whereas it showed cytoplasmic accumulation or clustering around dilated intracellular vesicles in HP lacking FOXA2 (Fig. 2D).

Furthermore, the effect of FOXA2 loss on MH was assessed on day 21 of differentiation. RT-qPCR analysis showed a significant downregulation in the mRNA levels of key hepatocyte markers, including *FOXA1*, *FOXA3*, *HNF4A*, *TTR*, and *CPS1*; however, *AAT*, *CYP3A4*, *CYP26A1*, and *ALB* levels were significantly upregulated in the absence of FOXA2 (Fig. 2E). Increased intracellular ALB levels were further validated by Western blotting (Fig. 2F). In addition, immunostaining showed that the ALB expression was clustered around dilated vesicles in many cells lacking FOXA2 compared to normal expression pattern in WT-MH (Fig. 2G).

### Lack of FOXA2 induces ER stress and increases apoptosis in hepatic progenitors and increases proliferation in mature hepatocytes

**Fig. 3:**
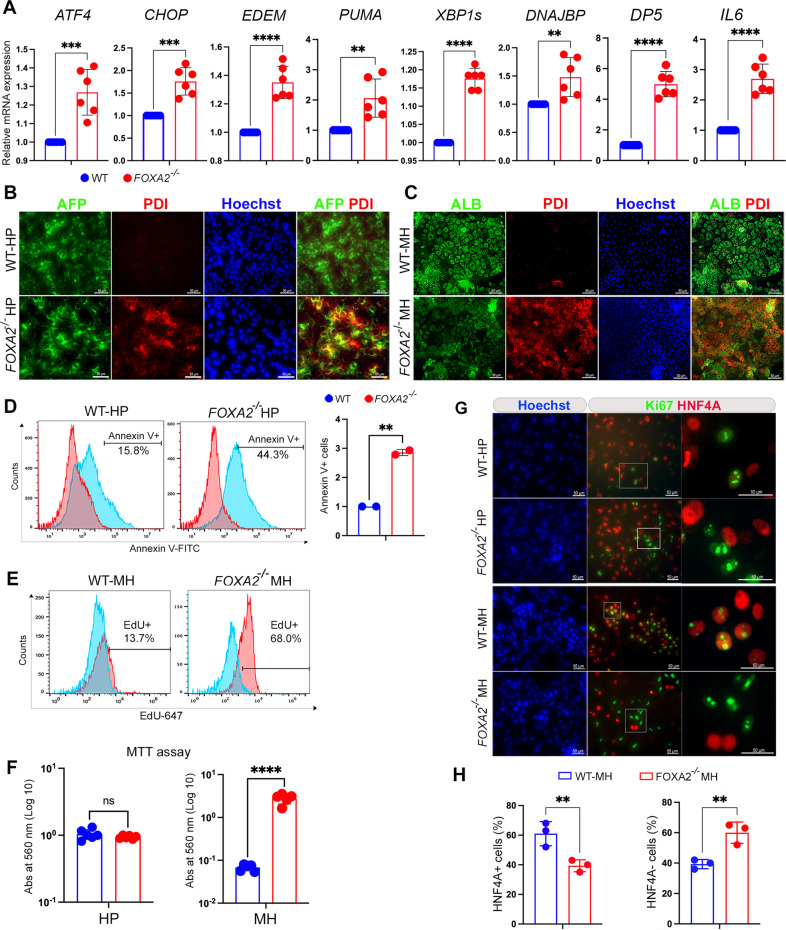
Effect of FOXA2 ablation on ER-stress markers, apoptosis, and proliferation in iPSC-derived hepatocytes. **A** RT-qPCR analysis showing the expression of ER stress genes, *ATF4*, *CHOP*, *EDEM*, *PUMA*, *XBP1s*, *DNAJBP*, *DP5*, and *IL6* in hepatic progenitors (HP) derived from *FOXA2*^*−/−*^iPSCs and WT controls (*n* = 6). **B** Immunofluorescence showing co-expression of ER stress marker, PDI, with AFP in FOXA2^−/−^HP (**B**) and with ALB in FOXA2^−/−^MH (**C**) compared to WT controls. **D** Flow cytometry analysis of cell apoptosis (Annexin V + cells) showing a significant increase in apoptosis in HP derived from FOXA2^−/−^iPSCs compared to those derived from WT-iPSCs (*n* = 2). **E** Flow cytometry analysis of EdU incorporation showing a dramatic increase in cell proliferation (EdU + cells) in mature hepatocytes (MH) derived from FOXA2^−/−^iPSCs in comparision to those derived from WT-iPSCs (*n* = 3). **F** Relative MTT levels in FOXA2^−/−^ HP and FOXA2^−/−^ MH relative to WT controls, quantified by measuring absorbance at 560 nm (*n* = 5). **G** Double immunofluorescence staining for Ki67 (proliferation marker) and HNF4A (hepatic marker) in FOXA2^−/−^HP and FOXA2^−/−^MH relative to WT controls. Squares indicate cells magnified in the right panels. Note clear distinction between Ki67 and HNF4A in HP and MH lacking FOXA2. **H** Percentage of HNF4A+ (hepatocytes) and HNF4A− (non-hepatocytes) cells in FOXA2^−/−^ MH compared to WT controls. The total number of cells was determined using Hoechst staining (*n* = 3). The data are presented as mean ± SD. *p < 0.05, **p < 0.01, ***p < 0.001.

Previous studies reported that lack of *Foxa2* gene in rodent liver induces ER stress [9]. Taken together with the observed defects in distribution of AFP and ALB in HP and MH derived from FOXA2^−/−^iPSCs suggest that FOXA2 loss may lead to a similar phenotype in human hepatocytes. Therefore, we sought to examine whether the absence of FOXA2 influences ER stress signaling by evaluating mRNA expression of key markers for the activation of unfolded protein response (UPR). We found that HP lacking FOXA2 showed a significant upregulation in the mRNA levels of ER stress markers, *ATF4*, *CHOP, EDEM, PUMA*, *XBP-1s, DNAJBP, DP*5, and *IL6* (Fig. 3A; Supplementary Fig. 4). Furthermore, co-staining of ER stress marker, PDI, with developmental markers, AFP and ALB, showed a marked increase in the PDI co-expression with AFP and ALB in HP and MH, respectively in the absence of FOXA2 compared to WT controls (Fig. 3B, C).

To investigate if the FOXA2^−/−^HP under ER-stress are more prone to cell death, apoptosis assay was performed on HP. Flow cytometry analysis showed a significant increase in the Annexin V + cells in HP lacking FOXA2 compared to WT (Fig. 3C). On the other hand, there was no significant change in cell death at the MH stage (Supplementary Fig. 4).

To evaluate the effect of FOXA2 loss on cell proliferation and metabolic activity, EdU and MTT assays were conducted. Flow cytometry quantification of the EdU incorporation showed a significant increase in EdU+ cells in MH lacking FOXA2 compared with WT controls; however, there were no significant changes between FOXA2^−/−^HP and WT controls (Fig. 3E). Consistent with these results, MTT assays showed a significant increase in metabolic activity of FOXA2^−/−^MH compared with WT controls, while there were no changes observed in HP (Fig. 3F). To determine nature of the proliferative cells, we visualized the expression of the Ki67, the proliferation marker, using immunostaining. Interestingly, our immunostaining results showed that the majority of the Ki67+ cells were co-localized with the hepatic marker, HNF4A, in WT controls of HP and MH (Fig. 3G). In contrast, most of the Ki67+ cells were not co-localized with HNF4A at both HP and MH lacking FOXA2 (Fig. 3G), suggesting that the increase in cell proliferation noticed at the MH stage is due to activation of non-hepatocyte cells. To determine the proportion of the hepatocytes and non-hepatocytes in MH, the HNF4A+ and HNF4A− cells were quantified. We found that the percentage of HNF4A+ (hepatocytes) was around 61% and 40% in WT-MH and FOXA2^−/−^MH, respectively, while the percentage of HNF4A− (non-hepatocytes) was around 39% and 60% in WT-MH and FOXA2^−/−^MH, respectively (Fig. 3H). This indicates that the absence of FOXA2 may activate the non-hepatocytes at the expense of the hepatocytes at the MH stage.

### FOXA2 ablation negatively impacts the function of iPSC-derived hepatocytes

**Fig. 4:**
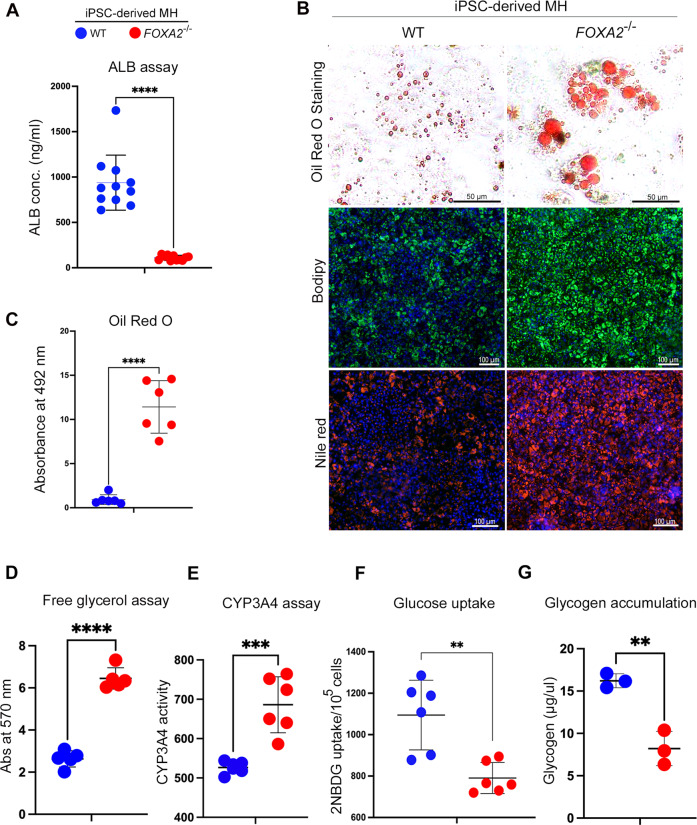
Loss of FOXA2 impairs functions of iPSC-derived hepatocytes. **A** ELISA quantification of ALBUMIN (ALB) concertation secreted from FOXA2^−/−^MH compared to WT controls (*n* = 11). **B** Representative images showing staining of MH derived from *FOXA2*^*−/−*^PSCs and WT-iPSCs with Oil Red O, BODIPY, and Nile red. **C** Oil Red O, free glycerol (**D**), and CYP3A4 activity (**E**) were measured in FOXA2^−/−^MH compared to WT controls (*n* = 6). **F** Relative glucose uptake in FOXA2^−/−^MH relative to WT controls (*n* = 6). **G** Relative glycogen accumulation in FOXA2^−/−^MH relative to WT controls (*n* = 3). Data are represented as mean ± SD; *p < 0.05, **p < 0.01, ***p < 0.001.

We next sought to assess the effect of FOXA2 loss on the hepatocyte functions by performing several assays on iPSC-derived MH. Although we found that mRNA and protein levels of ALB were significantly upregulated in the absence of FOXA2, we assessed the ability of MH lacking FOXA2 to produce ALB from hepatocyte to culture medium. ALB assay showed a significant reduction in ALB concentrations in the culture media of FOXA2^−/−^MH compared with WT controls (Fig. 4A, Supplementary Fig. 5), indicating a defect in ALB secretion due to FOXA2 ablation. Furthermore, the effect of elevated ER stress on lipid metabolism was explored, as ER is regarded to be the principal site of lipid metabolism [10]. This was accomplished by staining the intracellular lipid using Oil Red O, BODIPY, and Nile red (Fig. 4B). The staining showed a remarkable increase in the levels of Oil Red O, BODIPY, and Nile red in FOXA2^−/−^MH compared with WT controls, indicating increased lipid accumulation in the absence of FOXA2 (Fig. 4B). Quantification of Oil Red O staining showed a significant increase in lipid accumulation in FOXA2^−/−^MH compared with WT controls (Fig. 4C, Supplementary Fig. 5). Effect of increased lipid accumulation on resultant lipolysis was evaluated by quantifying free glycerol, as free glycerol along with free fatty acid is a product of triglyceride (TG) lipolysis. Our results showed an increase in glycerol levels in the absence of FOXA2 (Fig. 4C), indicating an upregulation in lipolysis to alleviate the burden of increased lipid accumulation. Furthermore, the effect on internal CYP3A4 activity in reaction to increased lipid accumulation was evaluated, as CYP3A4 is found to play a significant role in detoxifying hepatocytes from increased lipid-mediated toxicity [11]. Intracellular CYP3A4 protein activity levels were quantified by measuring its enzyme activity. We noticed a dramatic increase in CYP3A4 levels in FOXA2^−/−^MH in comparison to WT controls (Fig. 4D), suggesting that the absence of FOXA2 increases CYP3A4 levels to overcome the toxicity elicited by increased lipid accumulation.

Furthermore, the effect of FOXA2 deficiency on the glucose uptake potential of hepatocytes was measured, as hepatocytes account for one of the main tissues for insulin-mediated glucose uptakes [12]. Our analysis showed a significant decrease in glucose uptake in FOXA2^−/−^MH, suggesting that FOXA2 plays an important role in glucose metabolism in human hepatocytes (Fig. 4E). Effect of reduced glucose uptake on intracellular glycogen storage was examined, as glycogen is the principal reservoir for short-term storage of glucose by the process of glycogenolysis [13]. Our results indicated a significant reduction in glycogen accumulation in FOXA2^−/−^MH (Fig. 4F), thus aligning with the predicted consequence seen upon finding reduced glucose uptake.

### Transcriptome-wide alterations in hepatic progenitors and mature hepatocytes in the absence of FOXA2

**Fig. 5:**
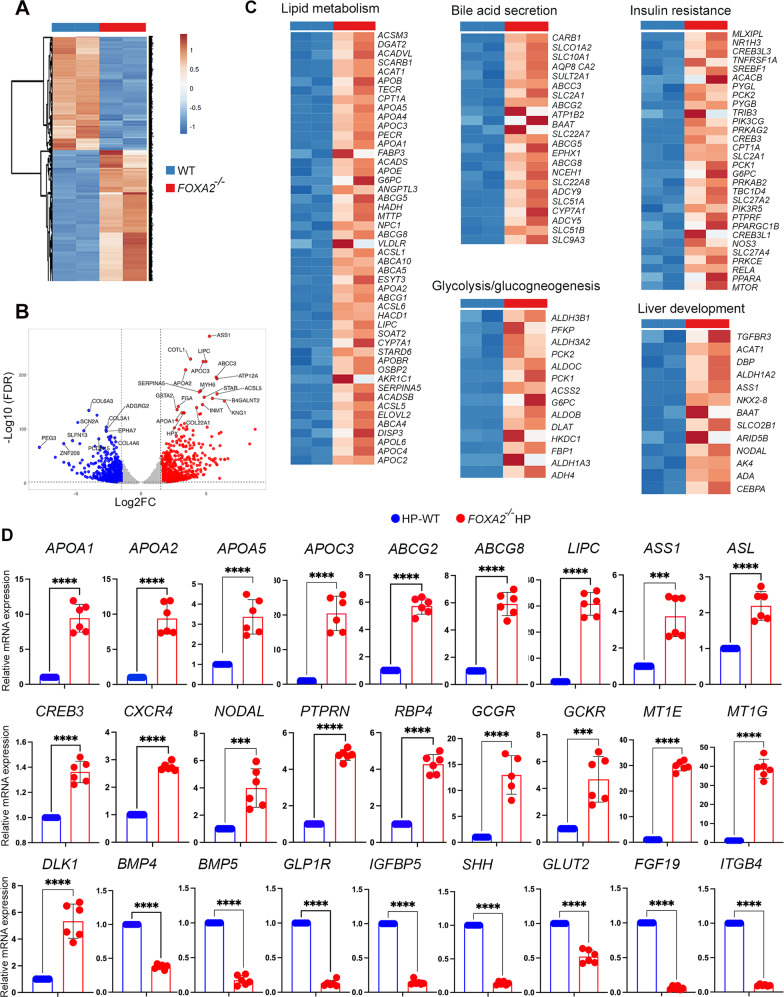
RNA-seq analysis of hepatic progenitors derived from WT and FOXA2^−/−^ iPSCs. **A** A clustering heatmap of differentially expressed genes (DEGs) in hepatic progenitors (HP) derived from WT and FOXA2^−/−^iPSCs. **B** Volcano plot of the differential gene expression between WT and FOXA2^−/−^HP (FDR < 0.05). Blue dots indicate downregulated genes, while red dots represent upregulated genes in FOXA2^−/−^HP. **C** Heatmaps of the key pathway-associated DEGs in FOXA2^−/−^HP compared to WT-HP (*p*-value < 0.05 and Log2 FC > 1.0). **D** Validation of RNA-seq results using RT-qPCR for the upregulated and downregulated genes (*n* = 6). Data are represented as mean ± SD; *p < 0.05, **p < 0.01, ***p < 0.001.

**Fig. 6:**
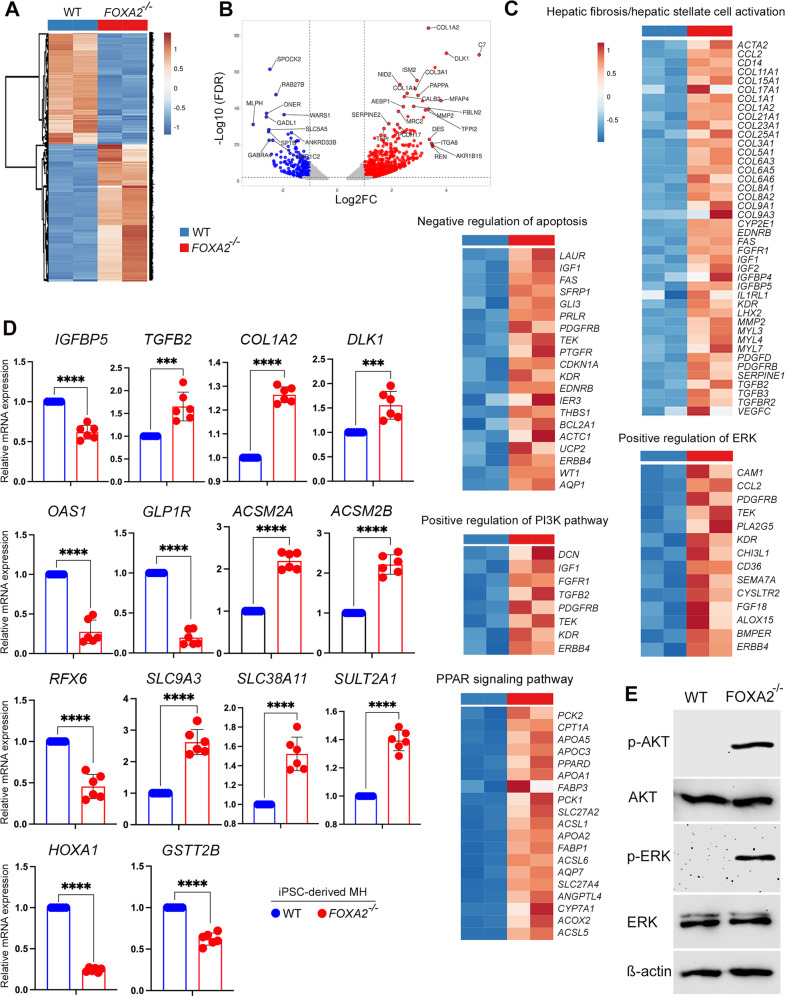
RNA-seq analysis of mature hepatocytes derived from WT and FOXA2^−/−^iPSCs. **A** A clustering heatmap of differentially expressed genes (DEG) in mature hepatocytes (MH) derived from WT and FOXA2^−/−^iPSCs. **B** Volcano plot of the differential gene expression between WT and FOXA2^−/−^MH (FDR < 0.05). Blue dots indicate downregulated genes, while red dots represent upregulated genes in FOXA2^−/−^MH. **C** Heatmaps of the key pathway-associated DEGs in FOXA2^−/−^MH compared to WT-MH (*p*-value < 0.05 and Log2 FC > 1.0). **D** Validation of RNA-seq results using RT-qPCR for the upregulated and downregulated genes (*n* = 6). **E** Western blotting of phosphorylated AKT (p-AKT) and phosphorylated ERK (p-ERK) showing a dramatic increase in p-AKT and p-ERK in FOXA2^−/−^MH compared to WT-MH. Data are represented as mean ± SD; *p < 0.05, **p < 0.01, ***p < 0.001.

To get an insight into the global changes in gene expression in the absence of *FOXA2*, we performed genome-wide RNA sequencing (RNA-seq) in HP and MH. We identified 2608 and 1122 differentially expressed genes (DEGs) in HPs and MHs, respectively (Figs. 5, 6). The most significantly altered genes are represented in Supplementary Tables 4–7. Of those DEGs genes, 1182 genes were significantly downregulated (Log2 FC < −1.0; *p*-value < 0.05) and 1426 were significantly upregulated (Log2 FC > 1.0; *p*-value < 0.05) in HPs lacking *FOXA2* in comparison to WT (Fig. 5A, B). To identify the enriched pathways, we used the ingenuity pathway analysis (IPA) and DAVID for further analysis on the DEGs. The biological pathways of the upregulated DEGs between WT and FOXA2^−/−^HP were mainly enriched in” lipid metabolism”, “bile acid secretion”, “PPAR signaling pathway”, “glycolysis/gluconeogenesis”, “insulin resistance”, “FOXO signaling pathway”, and “liver development” (Fig. 5C). However, the downregulated DEGs were mainly associated with “BMP signaling”, “hepatocyte differentiation”, and “cell cycle”. RT-qPCR was used to validate several DEGs associated with those pathways. In line with RNA-seq results, RT-qPCR analysis showed significant upregulation of *APOA1, APOA2*, *APOA5, APOC3, ABCG2, ABCG8, LIPC, ASS1, ASL, CREB3, CXCR4, NODAL, PTPRN, RBP4, GCGR, GCKR, MT1E, MT1G*, and *DLK1* (Fig. 5D). On the other hand, several genes were significantly downregulated, such as *BMP4, BMP5*, *GLP1R*, *IGFBP5*, *SHH*, *GLUT2*, *FGF19*, and *ITGB4* (Fig. 5D).

Furthermore, in MH, of 1122 DEGs genes, 254 genes were significantly downregulated (Log2 FC < −1.0; *p*-value < 0.05) and 868 were significantly upregulated (Log2 FC > 1.0; *p*-value < 0.05) in FOXA2^−/−^MH in comparison to WT controls (Fig. 6A, B). The gene networks and pathways of the DEGs were enriched in “hepatic fibrosis/hepatic stellate cell activation”, “axonal guidance”, “negative regulation of apoptosis”, “positive regulation of PI3K pathway”, “positive regulation of ERK pathway”, “response to hypoxia”, and “platelet activation and degranulation” (Fig. 6C). Validation of selected DEGs using RT-qPCR showed significant upregulation of *IGFBP5, TGFβ2, COL1A2, DLK1, ACSM2A, ACSM2B, SLC9A3, SLC38A11*, and *SULT2A1* and significant downregulation of *OAS1*, *GLP1R*, *RFX6*, *HOXA1*, and *GSTT2B* (Fig. 6D). Interestingly, Western blotting analysis showed a dramatic upregulation in phosphorylated AKT and phosphorylated ERK proteins in hepatocytes derived from FOXA2^−/−^iPSCs, confirming the activation of PI3K and ERK signaling pathways in the absence of FOXA2 (Fig. 6E).

### FOXA2 overexpression reverses defective phenotypes in hepatocytes derived from FOXA2^−/−^ iPSCs

**Fig. 7:**
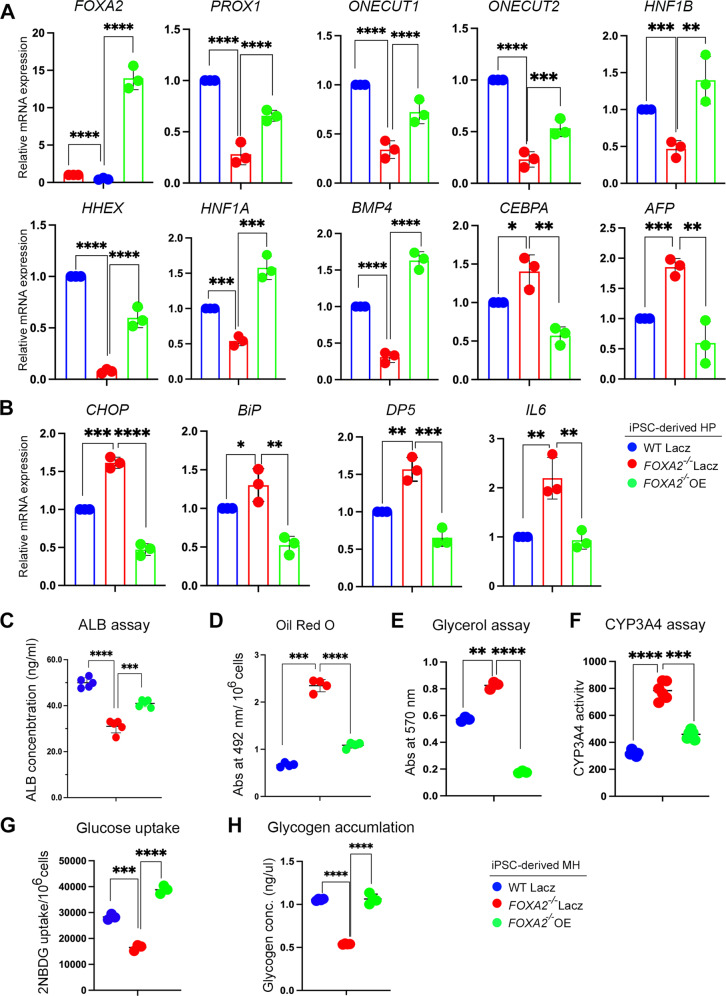
FOXA2 overexpression rescues developmental and functional defects of iPSC-derived hepatocytes lacking FOXA2. **A** RT-qPCR analysis for the expression of hepatic progenitor markers, *FOXA2*, *PROX1, ONECUT1*, *ONECUT1*, *HNF1B*, *HHEX*, *HNF1A*, *BMP4*, *CEBPA*, and *AFP* in HP derived from FOXA2^−/−^iPSCs and WT-iPSCs, 48 h after FOXA2 overexpression (*n* = 3). **B** RT-qPCR analysis for the expression of ER stress markers, *CHOP*, *BiP, DP5*, and *IL6* in HP derived from FOXA2^−/−^iPSCs and WT-iPSCs, 48 h after FOXA2 overexpression (*n* = 3). **C** ALBUMIN (ALB) ELISA assay showing ALB levels measured in culture media of mature hepatocytes (MH) derived from FOXA2^−/−^iPSCs, transfected with FOXA2 plasmid or empty vector (Lacz) at hepatic progenitor stage (*n* = 5). Quantification of Oil Red O staining (**D**) (*n* = 4), glycerol levels (*n* = 3) (**E**), CYP3A4 activity (**F**) (*n* = 6), glucose uptake (*n* = 3) (**G**), and glycogen accumulation *n* = 3) (**H**) in MH derived from FOXA2^−/−^iPSCs and WT-iPSCs, transfected with FOXA2 plasmid or empty vector at HP stage. Data are represented as mean ± SD; *p < 0.05, **p < 0.01, ***p < 0.001.

We next sought to examine the effect of ectopic FOXA2 (FOXA2-OE) on reversing defective phenotypes associated with FOXA2 loss in HP and MH. FOXA2 was overexpressed at day 8 of hepatocyte differentiation because this is the time point where FOXA2 was highly expressed during differentiation of WT controls (Fig. 1B). FOXA2-OE led to a significant upregulation in the mRNA expression levels of the developmental markers that were downregulated in FOXA2^−/−^HP, including *FOXA2*, *PROX1*, *ONECUT1*, *ONECUT2*, *HNF1B*, *HHEX*, *HNF41A*, and *BMP4* (Fig. 7A). It also resulted in a significant downregulation in the mRNA levels that were upregulated in *FOXA2*^*−/−*^HP, including *CEBPA* and *AFP* (Fig. 7A). Furthermore, the upregulated ER stress markers, *CHOP, BiP, DP5*, and *IL6* were significantly downregulated after FOXA2-OE in FOXA2^−/−^HP, indicating the reversal of the dysregulated mRNA levels in the presence of FOXA2 (Fig. 7B). In addition to that, the effect of transient FOXA2-OE conducted during HP stage was also assessed in MH at the functional level. Interestingly, the expression levels of FOXA2 in FOXA2^−/−^MH transfected at the HP stage was not significantly different from WT controls (Supplementary Fig. 6). Results showed a significant improvement in the levels of secreted extracellular ALB by showing a relative upregulation upon FOXA2-OE (Fig. 7C). Also, we noticed a reversal in excessive lipid accumulation by a relative decrease in Oil Red O staining upon FOXA2-OE in MH (Fig. 7D). Also, the increase in glycerol levels and CYP3A4 activity seen in the absence of FOXA2 was reversed due to FOXA2-OE (Fig. 7E, F). Moreover, the reduced glucose uptake and glycogen accumulation associated with FOXA2 absence was reversed upon FOXA2-OE (Fig. 7G, H).

## Discussion

Accumulating evidence from studies in rodents suggests the involvement of FOXA2 in hepatic function. However, its role in the development and functions of human hepatocyte is not fully understood. Liver-enriched TFs play essential roles in the development and metabolic functions of the liver [14–18]. Our results showed that the absence of FOXA2 resulted in significant alterations in the expression levels of the key TFs, led to a global change in the hepatic transcriptional program associated with developmental and functional defects. In contrast to our findings, deletion of Foxa2 in mouse liver does not change the expression levels of liver genes [19]; however, the expression of developmental genes is significantly altered in mouse liver lacking both Foxa1 and Foxa2 or triple Foxa1, Foxa2, and Foxa3 [3, 20], indicating the compensatory role played by the *Foxa* genes and suggest the difference between mouse and human models. Our results revealed that the loss of FOXA2 was not compensated by FOXA1 and FOXA3, particularly at the MH stage during hepatocyte development as previously reported in human [8] and rodent models [9, 19]. Taken together, our findings indicate that FOXA2 plays an essential role in hepatocyte and biliary development.

In hepatocytes, the main site of lipid metabolism is the ER; therefore, ER homeostasis is crucial for lipid metabolism. FOXA2 ablation resulted in a dramatic increase in lipid accumulation and a significant upregulation in the expression of genes associated with lipid metabolism and fat accumulation, such as apolipoproteins [21] and PPARG pathway genes [22]. Also, the loss of FOXA2 led to a significant upregulation in the expression of ER stress chaperones. This indicates the activation of unfolded protein response (UPR) pathway, which functions to maintain lipid homeostasis in hepatocytes [23]. The induction of ER stress leads to hepatic steatosis in primary hepatocytes [24] and saturated fatty acids can activate ER stress and cell death in hepatic cells [25]. Furthermore, the relationship between ER stress and lipolysis has been previously reported, lipolysis is elevated in response to ER stress conditions [26], supporting our finding of the increased lipolysis seen by higher glycerol release in the absence of FOXA2. Increased ERK1/2 activity has been reported to be associated with ER stress [27] and inhibiting ERK1/2 partially attenuated ER stress-mediated lipolysis [27]. In agreement with this result, we observed a marked increase in the p-ERK protein levels and upregulation in genes associated with ERK activation in hepatocytes lacking FOXA2, suggesting the involvement of ERK activation in ER stress activation. Interestingly, we noticed an upregulation in the expression of AFP and ALB in HP and MH lacking FOXA2 and their expressions were seen surrounding cytosolic vacuole-like structures in FOXA2-deficient cells. However, ALB concentration measured in the culture medium was dramatically reduced in MH lacking FOXA2, suggesting a defect in the ALB secretion. In mice, liver lacking Foxa2 showed a dilated ER due to ER stress [9], suggesting that the accumulation of these proteins in the dilated ER lumen is associated with ER stress. Taken together, our findings are in line with the role of FOXA2 in regulating lipid metabolism in mice [6] and suggest that activation of ER stress pathway in hepatocytes lacking FOXA2 may lead to hyperactivation of the lipid metabolism program.

Lipid-associated perturbations have been found to be a common hallmark among major metabolic disorders like obesity, diabetes, and insulin resistance. The molecular mechanism linking lipid-based abnormalities to metabolic disorders has been shown to be associated with insulin-mediated FOXA2 repression in obese and high-fat diet states, leading to elevated triglycerides and reduced plasma high-density lipoprotein (HDL) levels [28, 29]. This has been shown to be overcomed by nuclear reexpression of FOXA2 in an obese mice, resulting in elevated plasma HDL and reduced triglyceride levels, thus protecting mice from metabolic consequences of high-fat diet by FOXA2-mediated mechanism [28, 29]. In addition, it has been found that starvation can reactivate FOXA2 expression in liver cells [6]. In agreement with these mouse studies, we found that starvation enhanced FOXA2 expression in MH, indicating that FOXA2 can be reactivated in response to changes in metabolic conditions to protect the hepatocytes from defects related to FOXA2 functions.

Our RNA-seq analysis showed a significant upregulation in the expression of genes associated with bile acid secretion, such as *CYP7A1* and *ABCC3*, *ABCG2, ABCG5*, and *ABCG8* in hepatocytes lacking FOXA2 compared to WT controls. In the liver, the conversion of cholesterol to bile acids is initiated by CYP7A1 enzyme, which is predominant in humans [30]. Under normal conditions, ABCC3 is expressed at low level; however, it is elevated by ER stress [31] and in patients with obstructive cholestasis [32]. Elevation in bile acid transporters indicates that hepatocytes lacking FOXA2 acquire mechanisms for regulating intrahepatic bile acid levels in response to their elevated production. Also, our results showed that loss of FOXA2 significantly increased the expression and activity of CYP3A4, possibly in response to increased bile acid accumulation [11]. CYP3A4 enzyme is found to be active in detoxification and secretion of bile acid whereas, its induction by bile acid is essential for controlling bile acid-mediated toxicity [11]. Previous reports demonstrated that the ER stress and the UPR regulate bile acid homeostasis and increased lipolysis promotes bile acid production [31, 33]. These findings suggest that the induced ER stress and increased lipolysis associated with FOXA2 loss may lead to an increase in bile acid secretion.

The presence of ER stress, lipid accumulation, and hepatic cholestasis leads to hepatocellular injury activation and hepatic steatosis, enhancing the release of profibrogenic factors and stimulating the activation and propagation of HSCs, mainly through activation of the PI3K pathway [34, 35]. In this study, we observed a dramatic increase in the phosphorylated AKT protein and genes associated with PI3K-AKT pathway. In agreement with our findings, a recent study reported that silencing of FOXA2 in rat hepatic progenitors upregulates the expression of genes associated with PI3K pathway and increases AKT phosphorylation [36]. One of the key characteristics of activated HSCs is the secretion of large amounts of collagen, which is the main ECM protein in hepatic fibrosis [37, 38]. Of note, we found that at MH stage, loss of FOXA2 led to a significant increase in the expression of mRNA levels of genes associated with hepatic fibrosis and HSC activation, such as *PDGF* [39], *MMP2* [38], *TGFβ2*, *TGFβ3*, *TGFβR2* [40, 41], and *DLK1* [42] among others. These results suggest that loss of FOXA2 may induce HSC activation and hepatic fibrosis and the increased proliferation seen in the MH lacking FOXA2 may be due to the activation of the PI3K pathway and DLK1. Our immunostaining results confirmed that the increased proliferation seen at MH lacking FOXA2 is not related to hepatocytes, where most of the highly proliferative (Ki67+) cells were negative for HNF4A (hepatocyte marker) in the absence of FOXA2. Taken together, these findings indicate that loss of FOXA2 may promote the activation of the HSCs, which subsequently enhances hepatic fibrosis. These results are in line with those obtained from the patients, in which FOXA2 expression has been dramatically reduced in patients’ fibrotic livers [43].

**Fig. 8:**
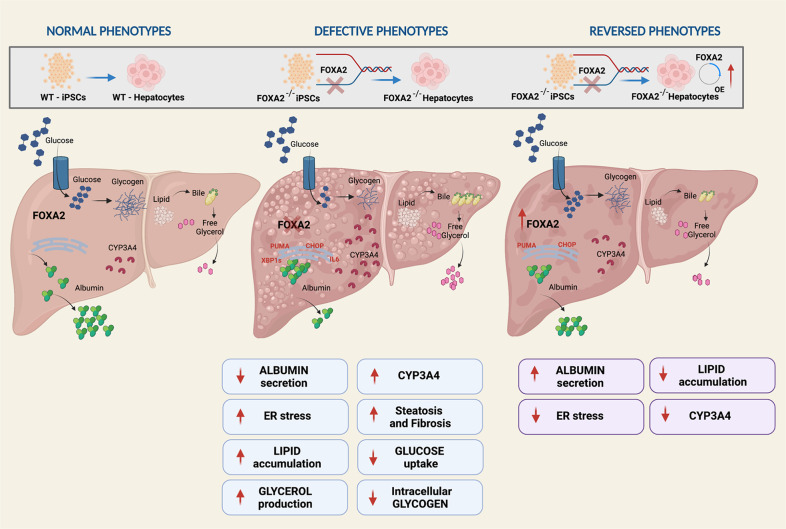
Graphical representation summarizing the role FOXA2 in the development and function of human hepatocytes. Absence of FOXA2 during the development of iPSC-derived hepatocytes alters developmental gene expression, reduced ALBUMIN secretion, induced ER stress, increased hepatic steatosis, and decreased glucose uptake and glycogen accumulation. However, overexpressing FOXA2 at a hepatic progenitor stage, reversed these defective phenotypes.

In conclusion, we have studied the role of FOXA2 in human hepatocyte development using the iPSC technology. The data presented here demonstrated that loss of FOXA2 induced ER stress and increased hepatic steatosis and bile acid toxicity in iPSC-derived hepatocytes (Fig. 8). Furthermore, we showed that hepatic developmental and functional genes were significantly dysregulated in the absence of FOXA2. Therefore, the appropriate expression of FOXA2 in human hepatocytes is essential for normal hepatocyte development and protects hepatic cells from ER stress, hepatic steatosis, and bile acid toxicity. This in vitro human model can be used as a platform to investigate the pathophysiological mechanisms associated with FOXA2-related hepatic defects.

## Materials and methods

### Culture and differentiation of iPSCs into hepatocytes

Two different iPSC lines (Ctr1-iPSCs and Ctr2-iPSCs) established in our laboratory were used in the current study [44]. *FOXA2* gene was knocked out in both iPSC lines using CRISPR/Cas9 as we recently reported [8] and cultured using Stemflex (ThermoFisher Scientific) media on Matrigel-coated plates (Corning). Wild-type (WT) and FOXA2^−/−^iPSCs were differentiated into hepatocytes using the previously published protocol [45] with slight modifications. For starvation experiments, MH were subjected to glucose free DMEM (ThermoFisher Scientific) for 6 h at 37 °C. Details of medium formulation are listed in Supplementary Table 1.

### Immunocytochemistry

Immunocytochemistry was performed as we previously reported [44, 46]. Cells were washed with DPBS (ThermoFisher Scientific) and fixed with 4% PFA (Sigma–Aldrich) at room temperature for 20 min, followed by washes with tris-buffered saline containing 0.5% Tween-20 (TBST) (Sigma–Aldrich) twice and permeabilized using phosphate buffered saline with 0.5% Triton (PBST) (Sigma–Aldrich) at room temperature for 20 mins. Cells were blocked with 6% bovine serum albumin (BSA) (Sigma–Aldrich) for two hours at room temperature and incubated with primary antibody at 4 °C overnight followed by incubation with secondary antibodies for 1 h at room temperature. Nuclei were stained with Hoechst33342 (ThermoFisher Scientific) and images were acquired using Olympus IX53 inverted fluorescence microscopy (primary and secondary antibody details are listed in Supplementary Table 2). For quantification of HNF4A+ and HNF4A− cells, attained images were fed into ImageJ and processed into 8-bit for threshold adjustments and dye quantifications.

### Western blotting

Cells from one well of a 6-well plate were collected for protein extraction using 300 µl RIPA buffer (ThermoFisher Scientific) containing protease inhibitor (ThermoFisher Scientific; Cat#: J61852.XF) at a final concentration of 1X, and protein concentration was measured using Pierce BCA kit (ThermoFisher Scientific). 20 µg of protein was mixed with 4X laemmili (Bio-Rad) at a final concentration of 1X and denatured at 95 °C for 5 min and ran on 10% gel at 150 volts for 60 min. Gel was transferred to nitrocellulose membrane with pore size of 0.2 µM at 30 volts for 1 h, followed by blocking in 10% skimmed milk in TBST for 2 h at room temperature, followed by incubation with primary antibody overnight at 4 °C. Membrane was washed with TBST and incubated with secondary antibody for 1 h at room temperature, followed by washes with TBST. Membrane was developed using chemiluminescent substrate (ThermoFisher Scientific) (antibody details are listed in Supplementary Table 2).

### Total RNA extraction, PCR, and RT-qPCR analyses

1 × 10^6^ cells were collected using 1000 µl TRIzol Reagent (Life Technologies) and subjected to RNA extraction kit (Norgen Biotech Corp). cDNA was synthesized from 1 µg of RNA in a reaction volume of 20 µl using Superscript IV First Strand Synthesis System (ThermoFisher Scientific). Real-time PCR (qPCR) was performed using SYBR Green Master Mix (ThermoFisher Scientific), at 10 µl reaction volume for accessing relative gene expression. GAPDH was used as a loading control (primer details are listed in Supplementary Table 3).

### Differential gene expression analysis

Strand-specific RNA-seq libraries were prepared using the TruSeq Stranded mRNA Library kit (Illumina) then sequenced on an Illumina HiSeq 4000 at a minimum of 20 million read pairs (2 × 75) per sample. The raw reads were quality-controlled using FastQC v.0.11.8, and the low-quality bases (Phred quality score <30) and adaptor contamination (if present) were removed by Trimmomatic v.0.36 [47], using the parameters ‘LEADING:3 SLIDINGWINDOW:4:20 MINLEN:25’. The high-quality reads were mapped by STAR v.2.7.2b [48] against the Homo Sapiens reference genome (ftp://ftp.ensembl.org/pub/release99/fasta/homo_sapiens/dna/Homo_sapiens.GRCh38.dna_sm.primary_assembly.fa.gz). The uniquely-mapped reads aligned to exons were counted with HTSeq v.0.9.1 [49], then tested by the DESeq2 R package v.1.26.0 [50] for the presence of differentially expressed genes (DEGs). All genes with a false discovery rate (FDR) less than 0.5 were considered DEGs regardless of their fold-change (FC) value. The raw and processed sequencing data associated with this experiment are deposited in the Gene Expression Omnibus (GEO) under the accession number GSE190763.

### Glucose uptake

Glucose uptake assay was performed as we recently reported [44]. Cells were dissociated post-endoderm and plated on a 96-well plate. On the day of assay, cells were washed with DPBS twice to remove residual media and incubated in DMEM- NO glucose for 5 h at 37 °C. This was followed by addition of 2[N-(7-nitrobenz-2-oxa-1,2-diaxol-4-yl) amino]-2-deoxyglucose (2-NBDG) at a final concentration of 150 μg/ml for 1.5 h at 37 °C. 2-NBDG treatment was removed by centrifugation of plate at 400 rpm and removal of supernatant. This was followed by washes with cell-based assay buffer (Cayman) twice and quantification of emitted fluorescence at 485/535 nm.

### Assessment of apoptosis

Cells were collected and dissociated using TryplE (ThermoFisher Scientific) and washed twice using DPBS (ThermoFisher Scientific) followed by suspension in cold 1X binding buffer (BD Biosciences) and incubation with Annexin-V for 15 min at room temperature. Stained cells were quantified for Annexin-V expression using BD Accuri C6 flow cytometer and analyzed using flowjo software.

### MTT assay

Cells were dissociated post-endoderm and plated on a 96-well plate. On the day of assay, cells were washed with DPBS twice. MTT solution was prepared in the differentiation basal media by dissolving MTT at a final concentration of 0.5 mg/ml and sterilized using 0.2 µM syringe filter. Prepared MTT was added as 100 µl/ well and incubated at 37 °C for 5 h. Media was aspirated and produced formazon was dissolved by adding 100 µl/well DMSO. Dissolved formazon was collected and quantified at 570 nm, while using DMSO as a blank.

### EDU assay

Mature hepatocytes were incubated with 20 μM EdU in differentiation media for 4 h at 37 °C. EdU was removed and cells were washed with DPBS and dissociated with TryplE. Cells were fixed with 4% PFA for 20 min at room temperature and washed with DPBS. Cells were blocked with 3% BSA, overnight at 4 °C. Fixed cells were used for EdU quantification as per the manufacturer’s instructions (Abcam).

### Liver function assays

#### Albumin ELISA

Cells at day 21 of hepatic differentiation were cultured for 48 h without changing media and resultant supernatant was used for carrying Albumin ELISA using manufacturer’s instructions (Bethyl Laboratories).

#### Free glycerol assay

Cells at day 21 of hepatic differentiation were washed with PBS twice and incubated with Kreb’s ringer buffer (NaCl 129 mM, KCL 4.8 mM, CaCl_2_ 2.5 mM, MgSO_4_ 1.2 mM, Na_2_HPO_4_ 1 mM, KH_2_PO_4_ 1.2 mM, NaHCO_3_ 5 mM, HEPES 10 mM, BSA 0.10%) for 3.5 h at 37 °C. Conditioned Kreb’s buffer was collected and used for glycerol quantification as per manufacturers instruction (Abcam).

#### Glucose uptake

Glucose uptake assay was performed as we recently reported [44]. Cells were washed with PBS and incubated in DMEM- NO glucose for 5 h at 37 °C. This was followed by addition of 2[N-(7-nitrobenz-2-oxa-1,2-diaxol-4-yl) amino]-2-deoxyglucose (2-NBDG) at a final concentration of 150 μg/ml for 1.5 h. This was followed by washes with cell-based assay buffer (Cayman) and quantification of emitted fluorescence at 485/535 nm.

#### Glycogen assay

Cells at day 21 of hepatic differentiation were washed with PBS and collected using trypsin. Cell pellet was resuspended in ice-cold water to disrupt cell membrane. Collected lysate was sonicated thrice, denatured at 95 °C for 5 min and spinned at 14,000 rpm for 30 min. Lysate was further used for glycogen quantification as per manufacturer’s instructions (Sigma-Aldrich).

#### P450-GLO CYP3A4 assay

Cells at day 21 of hepatic differentiation were washed with PBS and incubated with 50 μM dexamethasone for 48 h at 37 °C, followed by incubation with 6’-pentafluro-benzyl ether (PFBE) at 50 μM concentration for 24 h. Conditioned media was removed and used for CYP450 quantification as per manufacturers instruction (Promega).

#### Oil Red O quantification

Oil Red O staining and quantification was performed as previously reported [51]. Briefly, working oil red solution was prepared by mixing 6 parts 0.35% oil red (Sigma–Aldrich) in 4 parts ddH_2_O. Cells at day 21 of differentiation were washed with PBS and fixed with 4% PFA for 15 min at RT. Cells were further incubated with 60% isopropanol for 5 min and then allowed to air dry. Cells were then incubated with working oil red solution for 20 min at RT. Stained cells were washed with water 3–5 times and used for imaging. For dissolving oil red, cells were incubated with 100% isopropanol on orbital shaker, for 10 min at RT. Dissolved oil red was removed and quantified at 492 nm, while using 100% isopropanol as a control.

#### Nile red and BODIPY staining

Mature hepatocytes were stained for Nile Red (Abcam) and BODIPY (ThermoFisher Scientific) according to the manufacturer’s instructions.

### Overexpression of FOXA2

At the end of day 7, dissociated cells using ACCUATSE (Stem Cell Technologies) were cultured on Matrigel-coated plates in differentiation media supplemented with 10 µM Y27632. After 24 h, the cells were transfected either with FOXA2 plasmid (clone HsCD00330288 in pLenti6.2/v5-DEST; DNASU Plasmid Repository, Arizona State University, Tempe, AZ) or empty vector using Lipofectamine 3000 (Thermofisher Scientific). Cells were collected for RNA and protein extraction 48 h after transfection. Some experiments were continued until day 21 for functional analysis.

### Statistical analysis

Data are expressed as the mean ± standard deviation (SD). For most of the experiments, at least three biological replicates were used, and *p*-values was determined using two tailed Student’s *t* test. The graphs were prepared using Prism 8.

## Supplementary information

Supplementary Fig. 1

Supplementary Fig. 2

Supplementary Fig. 3

Supplementary Fig. 4

Supplementary Fig. 5

Supplementary Fig. 6

Supplementary Table 1

Supplementary Table 2

Supplementary Table 3

Supplementary Table 4

Supplementary Table 5

Supplementary Table 6

Supplementary Table 7

Western Original Data File

Reproducibility checklist

## Data Availability

The accession number for the RNA-seq data reported in this paper is GSE190763.
