# Supplementary material for: Loss of FOXA2 induces ER stress and hepatic steatosis and alters developmental gene expression in human iPSC-derived hepatocytes
Source: Cell Death Dis. 2022 Aug 16;13(8):713. doi: 10.1038/s41419-022-05158-0 (PMC9381545; doi:10.1038/s41419-022-05158-0)
Supplement: Supplementary file 1 — Supplementary Fig. 1 [file 41419_2022_5158_MOESM1_ESM.pdf]

Supplementary Fig. 1

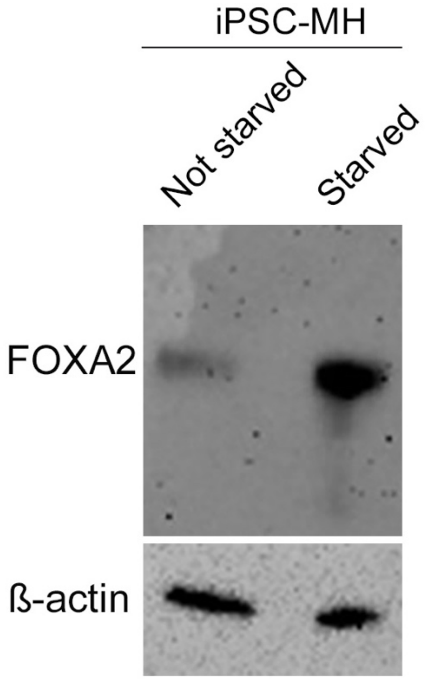

Supplementary Fig. 1. Effect of starvation on FOXA2 expression in iPSC-derived mature hepatocytes. iPSC-derived MH cultured under starved and normal conditions was examined for FOXA2 expression using Western blotting. FOXA2 protein was dramatically induced in the starved MH in comparison those cultured under normal conditions.
