# Supplementary material for: Loss of FOXA2 induces ER stress and hepatic steatosis and alters developmental gene expression in human iPSC-derived hepatocytes
Source: Cell Death Dis. 2022 Aug 16;13(8):713. doi: 10.1038/s41419-022-05158-0 (PMC9381545; doi:10.1038/s41419-022-05158-0)
Supplement: Supplementary file 2 — Supplementary Fig. 2 [file 41419_2022_5158_MOESM2_ESM.pdf]

Supplementary Fig. 2

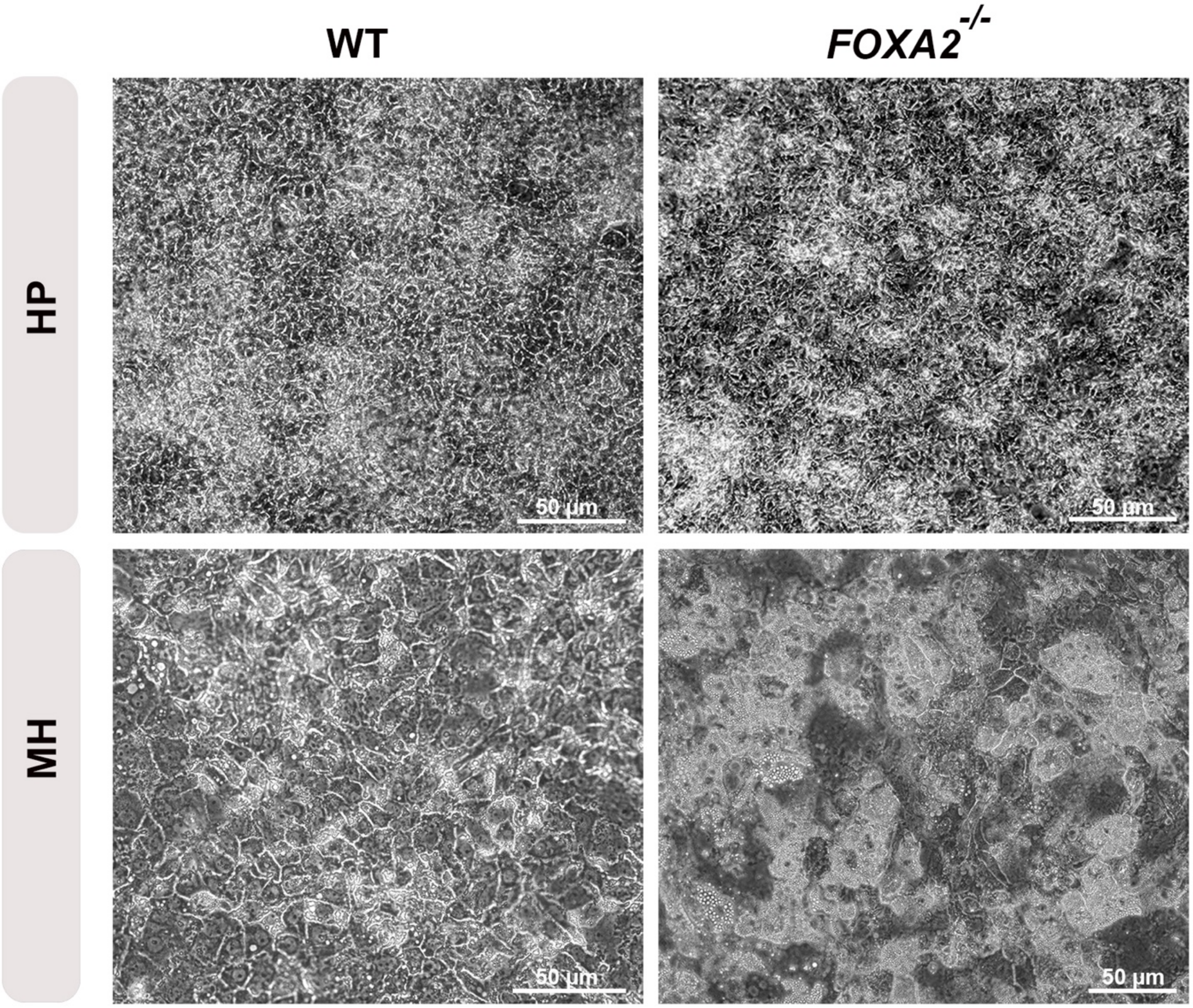

Supplementary Fig. 2. Morphology of hepatic progenitors and mature hepatocytes derived from WT and FOXA2$^{-/-}$ iPSCs. Representative bright field images showing the differences in the morphology between different conditions at hepatic progenitors (HP) and mature hepatocytes (MH) stages. Note the morphological changes between HP and mature MH. Furthermore, there was clear alterations in the morphology of FOXA2$^{-/-}$MH compared to WT-MH. Scale bars = 50 µm.
