# Supplementary material for: Loss of FOXA2 induces ER stress and hepatic steatosis and alters developmental gene expression in human iPSC-derived hepatocytes
Source: Cell Death Dis. 2022 Aug 16;13(8):713. doi: 10.1038/s41419-022-05158-0 (PMC9381545; doi:10.1038/s41419-022-05158-0)
Supplement: Supplementary file 3 — Supplementary Fig. 3 [file 41419_2022_5158_MOESM3_ESM.docx]

**Supplementary Fig. 3**


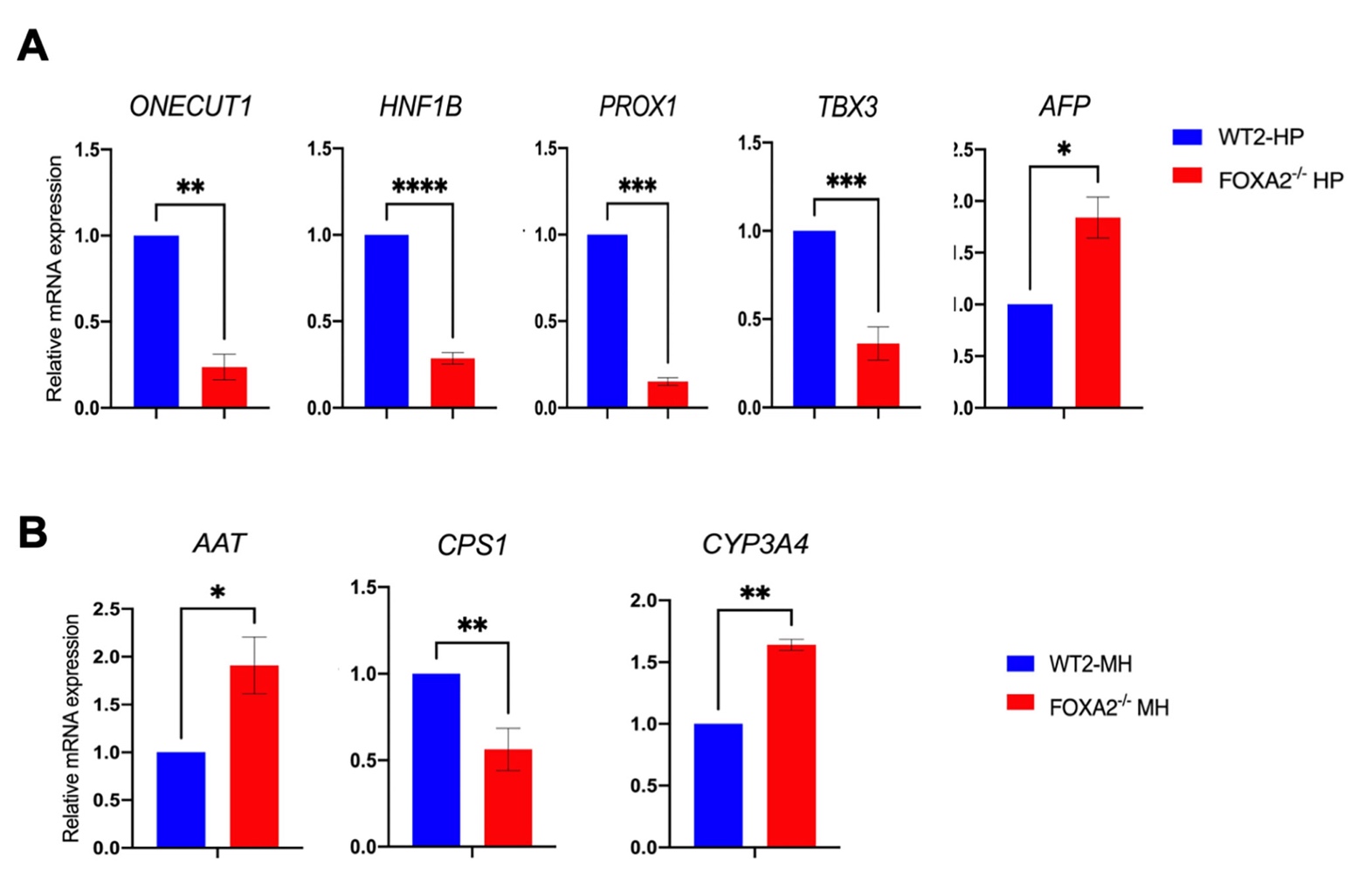


**Supplementary Fig. 3. Effect of FOXA2 loss on the expression of liver genes** in **hepatocytes derived from FOXA2^-/-^ iPSC line 2.** (A) RT-qPCR analysis showing the mRNA expression of hepatic progenitor (HP) markers*,* *ONECUT1*, *ONECUT2*, *HNF1B*, *PROX1*, *TBX3,* and *AFP* in FOXA2^-/-^ HP relative to wild type (WT) controls. (B) RT-qPCR analysis showing the mRNA expression of mature hepatocyte (MH) markers*,* *AAT*, *CPS1,* and *CYP3A4* in *FOXA2^-/-^* MH relative to WT controls. The data are presented as mean ±SD. **p* < 0.05, ***p* < 0.01, ****p* < 0.001.
