# Supplementary material for: Loss of FOXA2 induces ER stress and hepatic steatosis and alters developmental gene expression in human iPSC-derived hepatocytes
Source: Cell Death Dis. 2022 Aug 16;13(8):713. doi: 10.1038/s41419-022-05158-0 (PMC9381545; doi:10.1038/s41419-022-05158-0)
Supplement: Supplementary file 4 — Supplementary Fig. 4 [file 41419_2022_5158_MOESM4_ESM.docx]

**Supplementary Fig. 4**


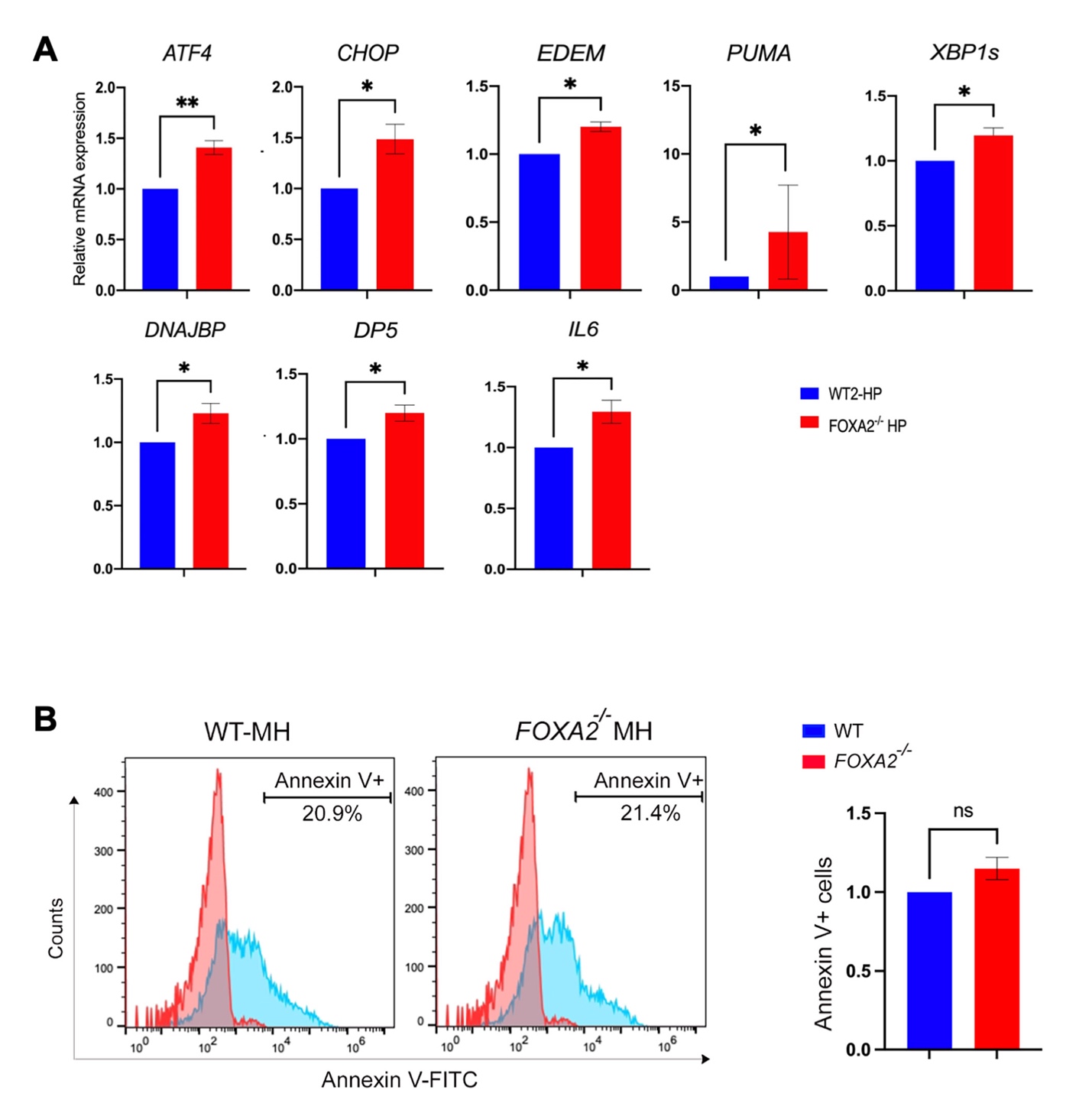


**Supplementary Fig. 4.** Loss of FOXA2 induces ER stress and apoptosis in hepatocytes derived from FOXA2^-/-^ iPSC line 2. (A) RT-qPCR analysis showing the mRNA expression of hepatic progenitor (HP) markers*,* *ONECUT1*, *ONECUT2*, *HNF1B*, *PROX1*, *TBX3,* and *AFP* in FOXA2^-/-^ HP relative to wild type (WT) controls. (B) Flow cytometry analysis of cell apoptosis (Annexin V+ cells) showing no significant difference in apoptosis between MH derived from FOXA2^-/-^iPSCs compared to those derived from WT-iPSCs (n=2). The data are presented as mean ±SD. **p* < 0.05, ***p* < 0.01, ****p* < 0.001.
