# Supplementary material for: Loss of FOXA2 induces ER stress and hepatic steatosis and alters developmental gene expression in human iPSC-derived hepatocytes
Source: Cell Death Dis. 2022 Aug 16;13(8):713. doi: 10.1038/s41419-022-05158-0 (PMC9381545; doi:10.1038/s41419-022-05158-0)
Supplement: Supplementary file 5 — Supplementary Fig. 5 [file 41419_2022_5158_MOESM5_ESM.docx]

**Supplementary Fig. 5**


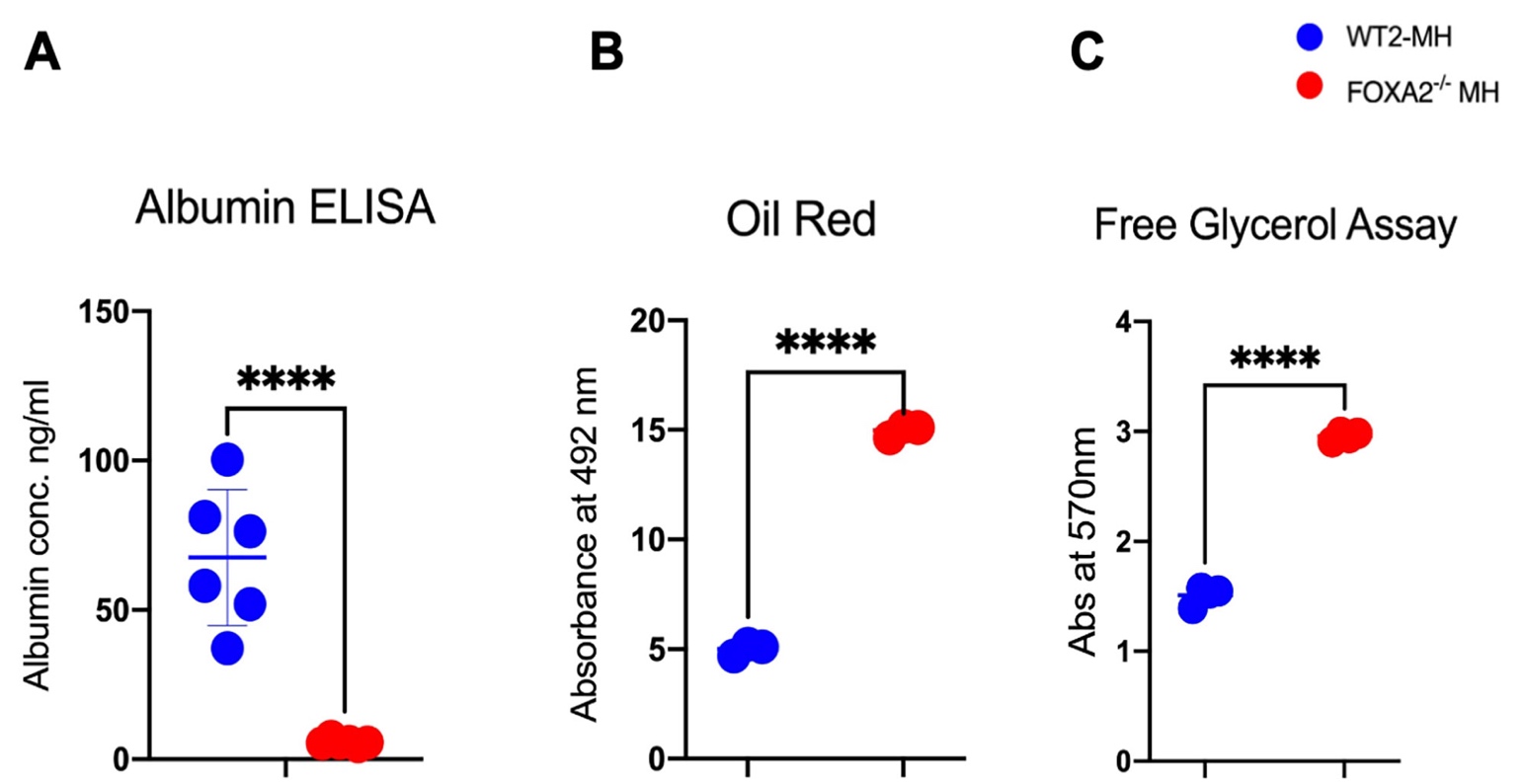


**Supplementary Fig. 5. Effect of FOXA2 knockout on the functions of hepatocyte derived from FOXA2^-/-^ iPSC line 2.** (A) ELISA quantification of ALBUMIN (ALB) concentration secreted from FOXA2^-/-^MH compared to WT controls. Quantification of Oil Red O staining (B) and measurement of free glycerol (C) in FOXA2^-/-^MH compared to WT controls. The data are presented as mean ±SD. **p* < 0.05, ***p* < 0.01, ****p* < 0.001.
