# Supplementary material for: Loss of FOXA2 induces ER stress and hepatic steatosis and alters developmental gene expression in human iPSC-derived hepatocytes
Source: Cell Death Dis. 2022 Aug 16;13(8):713. doi: 10.1038/s41419-022-05158-0 (PMC9381545; doi:10.1038/s41419-022-05158-0)
Supplement: Supplementary file 6 — Supplementary Fig. 6 [file 41419_2022_5158_MOESM6_ESM.pdf]

Supplementary Fig. 6

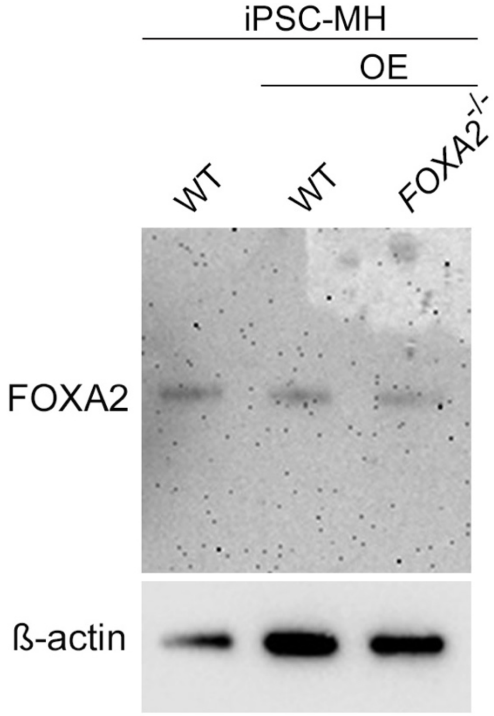

Supplementary Fig. 6. Western blotting for FOXA2 expression in mature hepatocytes (MH) derived from WT and FOXA2$^{-/-}$ hepatic progenitors (HP) transfected with FOXA2 plasmid. There was no significant difference in the FOXA2 protein levels between different conditions.
