# Supplementary material for: Loss of FOXA2 induces ER stress and hepatic steatosis and alters developmental gene expression in human iPSC-derived hepatocytes
Source: Cell Death Dis. 2022 Aug 16;13(8):713. doi: 10.1038/s41419-022-05158-0 (PMC9381545; doi:10.1038/s41419-022-05158-0)
Supplement: Supplementary file 7 — Supplementary Table 1 [file 41419_2022_5158_MOESM7_ESM.docx]

**Supplementary Table 1**: Media formulations for differentiation of iPSCs into hepatocytes

| **Stage** | **Basal Media** | **Cytokines** |
| --- | --- | --- |
| Stage 1 (Definitive endoderm)  Day 1 | MCDB 131+  1.5 g/l NaHCO_3_  2 mM glutamax  1% Pen/Strep  0.5% fatty acid-free BSA | 100 ng/ml Activin A  3 μM CHIR99021  0.25 mM Vitamin C  10 μM Y-27632 |
| Stage 1  (Definitive endoderm)  Day 2-4 | MCDB 131+  1.5 g/l NaHCO_3_  2 mM glutamax  1% Pen/Strep  0.5% fatty acid free BSA | 100 ng/ml Activin A  0.25 mM Vitamin C |
| Stage 2  (Posterior foregut)  Day 5 | MCDB 131+  1.5 g/l NaHCO_3_  2 mM glutamax  1% Pen/Strep  0.5% fatty acid free BSA | 10 ng/ml FGF2  30 ng/ml BMP4  2 μM Retinoic Acid  10 mM SB431542  10 μM Y-27632 |
| Stage 2  (Posterior foregut)  Day 6 | MCDB 131+  1.5 g/l NaHCO_3_  2 mM glutamax  1% Pen/Strep  0.5% fatty acid free BSA | 10 ng/ml FGF2  30 ng/ml BMP4  10 mM SB431542 |
| Stage 3  (Hepatic progenitors)  Day 7-8 | DMEM-F12  20% KOSR  1% NEAA  1% Glutamax  1% Penstrep | 10 ng/ml Activin A  30 ng/ml BMP4  1 μM Forskolin |
| Stage 3  (Hepatic progenitors)  Day 9 | DMEM-F12  20% KOSR  1% NEAA  1% Glutamax  1% Penstrep | 10 ng/ml Activin A  30 ng/ml BMP4  1μM Forskolin  1 μM Chir99021 |
| Stage 4  (Maturation)  Day 10-11 | IMDM  10% KOSR  0.5% NEAA  1% Glutamax  1% Pen/Strep | 10 ng/ml BMP4  10 ng/ml Oncostatin  10 mg/ml ITS  200 μg/ml Ascorbic Acid  0.1 μM Gamma Secretase Inhibitor  10 μM Dexamethasone  1 μM SB431542 |
| Stage 4  (Maturation)  Day 12-16 | IMDM  10% KOSR  0.5% NEAA  1% Glutamax  1% Pen/Strep | 10 ng/ml BMP4  10 ng/ml Oncostatin  10 mg/ml ITS  200 μg/ml Ascorbic acid  0.1μM Gamma secretase inhibitor  10 μM Dexamethasone |
| Stage 4  (Maturation)  Day 17-21 | IMDM  10% KOSR  0.5% NEAA  1% Glutamax  1% Pen/Strep | 10 mg/ml ITS  200 μg/ml Ascorbic Acid  0.1μM Gamma secretase inhibitor  10 μM Dexamethasone  1 μM Forskolin |
