# Supplementary material for: Loss of FOXA2 induces ER stress and hepatic steatosis and alters developmental gene expression in human iPSC-derived hepatocytes
Source: Cell Death Dis. 2022 Aug 16;13(8):713. doi: 10.1038/s41419-022-05158-0 (PMC9381545; doi:10.1038/s41419-022-05158-0)
Supplement: Supplementary file 8 — Supplementary Table 2 [file 41419_2022_5158_MOESM8_ESM.docx]

**Supplementary Table 2:** Antibodies used for Western blotting and immunofluorescence

| **Antibody** | **Catalog #** | **Company** | **Dilution** |
| --- | --- | --- | --- |
| Rabbit anti-FOXA2 | 3143 | Cell Signaling Technology | WB (1:5000)  IS (1:500) |
| Mouse anti-AFP | A8452 | Sigma Aldrich | WB (1:5000)  IS (1:4000) |
| Rabbit anti-HNF4A | 3113S | Cell Signaling Technology | IS (1:500) |
| Anti-CEBPA | Ab40761 | Abcam | IS (1:500) |
| Anti-ALBUMIN | A6684 | Sigma | WB (1:5000)  IS (1:500) |
| Anti-CYP3A4 | Ab135813 | Abcam | IS (1:500) |
| Anti-AAT | MAB1268 | R&D | IS (1:500) |
| Anti-CPS1 | Ab45956 | Abcam | IS (1:500) |
| Mouse anti-Ki67 | 561165 | BD | IS (1:500) |
| Anti-PD1 | 86163 | Cell Signaling Technology | IS (1:500) |
