# Supplementary material for: Loss of FOXA2 induces ER stress and hepatic steatosis and alters developmental gene expression in human iPSC-derived hepatocytes
Source: Cell Death Dis. 2022 Aug 16;13(8):713. doi: 10.1038/s41419-022-05158-0 (PMC9381545; doi:10.1038/s41419-022-05158-0)
Supplement: Supplementary file 9 — Supplementary Table 3 [file 41419_2022_5158_MOESM9_ESM.docx]

**Supplementary Table 3.** The list of primers used for qPCR analysis

| **Gene Name** | **Forward** | **Reverse** |
| --- | --- | --- |
| AFP | ACAGAGGAACAACTTGAGGCTGTC | AGCAAAGCAGACTTCCTGTTCCTG |
| PROX1 | AAAGTCAAATGTACTCCGCAAGC | CTGGGAAATTATGGTTGCTCCT |
| HNF1B | ACACACCTCCCATCCTCAAG | CATTTTAGCAGCCCTCCAAG |
| HNF6 | GGACCTCAAGATAGCAGGTTTAT | CAGAATGCAGGTGAGCTAAGT |
| CEBPA | CCACGCCTGTCCTTAGAAAG | CCCTCCACCTTCATGTAGAAC |
| GLUT2 | ATGAACTGCCCACAATCTCATA | GGACCAGAGCATGGTGATTAG |
| HHEX | GCGAGAGACAGGTCAAAACC | AGGGCGAACATTGAGAGCTA |
| ISL1 | CTGTGGACATTACTCCCTCTTAC | GCAACCAACACATAGGGAAATC |
| MT1E | GCTTGTTCGTCTCACTGGTG | TTGCAGGAGGTGCATTTG |
| MT1G | CCACTGCCTCTTCCCTTCTC | GAGACACCAGCGGCACAG |
| TCF7L2 | CGTAGACCCCAAAACAGGAA | TCCTGTCGTGATTGGGTACA |
| BMP4 | GACTACATGCGGGATCTTTAC | GGATGTTCTCCAGATGTTCTTC |
| ONECUT2 | ACTGTTCTCTGAGCATGCTAAG | AGCTTGGTTGACACCATACTG |
| ADAMTS20 | GTGTGTCAGAAAGTCAAATA | CTGGAATGTCTTCTTTCATC |
| HOXC4 | CCTCTCACCTTGTCCCTTGT | TAACAGTGGGAGGGAGAGGA |
| HOXB5 | TCCACAAATCAAGCCCTCCA | TAGCAACTGATAGTCCGGGC |
| GLI2 | AAGTCACTCAAGGATTCCTGCTCA | GTTTTCCAGGATGGAGCCACTT |
| ABCG2 | TTATCCGTGGTGTGTCTGGA | GATGATTGTTCGTCCCTGCT |
| CXCR4 | CACCGCATCTGGAGAACCA | GCCCATTTCCTCGGTGTAGTT |
| PTPRN | CCTACCAAGCAGAGCCAAAC3 | TGGTCATAGGGCAGGAAGTC |
| RBP4 | GCCTCTTTCTGCAGGACAAC | GCACACGTCCCAGTTATTCA |
| NODAL | GTCACCTTTTCCTTGGGCAG | ACCCTGGACATCTGCTTCTC |
| ONECUT3 | CGTCAGCAACTTCTTCATGAACG | CTCAGGCCTTGGAGAAAGTG |
| IGFBP5 | GTAGAACCTGCGAATTTCGAAG | AGAACAGGTAAGAGGCGTTG |
| BMP5 | ACAGGAAGGTTCTGTTGTGTG | GGCCTTAGAGGGCATTCTAATAG |
| CREB3 | TTTCCGTAGTTGTCCCAAATG | AAATCTCCACTTTCCTCTAGCAGG |
| CYP26A1 | GAAACCTTGCAGATGGTACTG | CGAACAGATGCGTCTTGTAGATG |
| PCK1 | CCTTCCCACTGGGAACACAAAC | CATTTCTGCAGAGTGCTGCTAAGG |
| ACSL5 | CACGTTAGAAAGCCTGACATG | TTCTTCCTGCCACACGAGTC |
| ZNF208 | TCCTGGGTATTGCTGCCTTT | TCCACCATCTCATGTCTCTTCA |
| PDGFA | GAGGTGATCGAGAGGCTGG | AAGAGGGCCAAGTAGACAGG |
| FGF19 | CTGACATGTTCTCTTCGCCC | CTCAAAGCTGGGACTCCTCA |
| SHH | CCGGCTTCGACTGGGTGTACTA | CGCCACCGAGTTCTCTGCTTT |
| ITGB4 | TGGAAGTACTGTGCCTGCTG | TGCATGTTGTTGGTGACCTT |
| TBX3 | CCCGGTTCCACATTGTAAGAG | GTATGCAGTCACAGCGATGAAT |
| APOA2 | GTTCGGAGACAGGCAAAGGA | TCAAAGTAAGACTTGGCCTCGG |
| GCKR | CATGTCGGTGTCCTTTAAT | CCTTCTTCAGTTCCTCAATC |
| APOA1 | ATGAAAGCTGCGGTGCTGACC | TCTTGCAGGGCCTATTTATGTC |
| LIPC | TGAAACCAGAGCCATTTGGAAG | GTCCGGATGATTGATTCGAATC |
| GCGR | AGGTGATGGACTTCCTGTTTGAG | TACTTGTCGAAGGTTCTGTTGC |
| APOC3 | CTTCATGCAGGGTTACATGAAG | TTTCAGGGAACTGAAGCCATC |
| APOA5 | AAGCAGAATGTCTGCTCTCTGTG | AAACGCTGAAAGAAGAGCCAGAG |
| ZNF208 | TCCTGGGTATTGCTGCCTTT | TCCACCATCTCATGTCTCTTCA |
| SULT2A1 | TCCTGGCCAACATGATGAAAC | CTGAGTTCAAACGATTCTCCTACC |
| PDGFA | GAGGTGATCGAGAGGCTGG | AAGAGGGCCAAGTAGACAGG |
| FGF19 | CTGACATGTTCTCTTCGCCC | CTCAAAGCTGGGACTCCTCA |
| SHH | GAAAGCAGAGAACTCGGTGG | GGAAAGTGAGGAAGTCGCTG |
| ASS1 | TGTGCTTATAACCTGGGATGG | GGAGCCTTTGCTGGACATAG |
| ASL | AGAAGTTCAACGCGTCCATTG | TTGTCTAGGCCATGGAGTATC |
| GLP1R | CCTGAAGTGGATGTATAGCACAG | GTACTGCATGAGCAGAAACAC |
| ONECUT2 | GCCATCTTCAAGGAGAACAAAC | CGTTCATGAAGAAGTTGCTGAC |
| CYP3A5 | CACAGAACACAGTTGAAGAAGG | TACGGGTCCCATATAGATAGAGG |
| HNF4A | CTGGAATTTGAGAATGTGCAGG | CTCGAGGCACCGTAGTGTTT |
| HNF1A | TGCAAGGAGTTTGGTTTGTG | CAGCTGGCTCAGTTTAGAAAC |
| SLC10A1 | CATGAACCTCAGCATTGTGATGAC | ATGCCTTTATAGGGCACCTTG |
| BST2 | CAATGTCACCCATCTCCTG | TGTAGTGATCTCTCCCTCAA |
| IFITM3 | CTACTCCGTGAAGTCTAGG | GATGACGATGAGCAGAATG |
| SLC16A3 | CATCACGGGGTTGGGTTTG | ACACAGGAAGACAGGGCTAC |
| IGF1 | AAGGAGGCTGGAGATGTATTG | TGTACTTCCTTCTGGGTCTTG |
| SLC38A11 | TTTGTGACAAGAGAGGTAAT | CAATCAGCAATGACACAAG |
| OAS1 | GGATTCTGCTGGCTGAAAG | GCTGGGTCTATGAGAGAAATG |
| HOXA1 | GCCCCTACGCGTTAAATCAG | GATGCTGGACCATGGGAGAT |
| CYCLIN D1 | GGCACAAGTCCTGGATGTTG | CCAGAAATGCACAGACCCAG |
| ALB | GTGAAACACAAGCCCAAGGCAACA | TCAGCCTTGCAGCACTTCTCTACA |
| AAT | AGGTGCCTATGATGAAGCGT | TGGCAGACCTTCTGTCTTCATT |
| TTR | CATGGGCTCACAACTGAGGA | TTGGCTGTGAATACCACCTCTG |
| CPS1 | TTTCAAAGTGGTGAGGACACTG | TCTTAGTTCCATCTTCCAGGAC |
| HNF4A | CTGGAATTTGAGAATGTGCAGG | CTCGAGGCACCGTAGTGTTT |
| FOXA2 | GGGAGCGGTGAAGATGGA | TCATGTTGCTCACGGAGGAGTA |
| TBX3 | CCCGGTTCCACATTGTAAGAG | GTATGCAGTCACAGCGATGAAT |
| GAPDH | ACGACCACTTTGTCAAGCTCATTTC | GCAGTGAGGGTCTCTCTCTTCCTCT |
