# Supplementary material for: Loss of FOXA2 induces ER stress and hepatic steatosis and alters developmental gene expression in human iPSC-derived hepatocytes
Source: Cell Death Dis. 2022 Aug 16;13(8):713. doi: 10.1038/s41419-022-05158-0 (PMC9381545; doi:10.1038/s41419-022-05158-0)
Supplement: Supplementary file 10 — Supplementary Table 4 [file 41419_2022_5158_MOESM10_ESM.docx]

**Supplementary Table 4. Top upregulated genes in hepatic progenitors derived from FOXA2^-/-^ iPSCs compared with WT controls**

| **Gene name** | **Gene description** | **Log2 FC** | ***p-*value** |
| --- | --- | --- | --- |
| APOA4 | apolipoprotein A4 | **8.746** | **0.0** |
| SDS | serine dehydratase | **8.331** | **9.33E-76** |
| KCNB1 | potassium voltage-gated channel subfamily B member 1 | **6.654** | **3.95E-48** |
| RASAL3 | RAS protein activator like 3 | **6.413** | **2.40E-46** |
| PTGS1 | prostaglandin-endoperoxide synthase 1 | **6.404** | **8.03E-47** |
| KNG1 | kininogen 1 | **6.378** | **7.93E-156** |
| CYP26A1 | cytochrome P450 family 26 subfamily A member 1 | **6.129** | **1.53E-97** |
| C11orf86 | chromosome 11 open reading frame 86 | **6.099** | **6.55E-47** |
| HMX1 | H6 family homeobox 1 | **6.048** | **2.25E-72** |
| GPR101 | G protein-coupled receptor 101 | **5.981** | **2.65E-95** |
| NLRC5 | NLR family CARD domain containing 5 | **5.821** | **1.94E-36** |
| ACSL5 | acyl-CoA synthetase long chain family member 5 | **5.811** | **5.53E-169** |
| ATP12A | ATPase H+/K+ transporting non-gastric alpha2 subunit | **5.808** | **2.23E-197** |
| CD22 | CD22 molecule | **5.783** | **8.31E-38** |
| ABCC3 | ATP binding cassette subfamily C member 3 | **5.746** | **5.44E-200** |
| F13B | coagulation factor XIII B chain | **5.687** | **2.12E-41** |
| ASPA | aspartoacylase | **5.687** | **3.02E-48** |
| MYH4 | myosin heavy chain 4 | **5.563** | **1.24E-46** |
| SULT1A2 | sulfotransferase family 1A member 2 | **5.551** | **7.17E-67** |
| ASB4 | ankyrin repeat and SOCS box containing 4 | **5.525** | **6.01E-44** |
| B4GALNT2 | beta-1,4-N-acetyl-galactosaminyltransferase 2 | **5.455** | **1.12E-160** |
| ADAMTS14 | ADAM metallopeptidase with thrombospondin type 1 motif 14 | **5.419** | **1.64E-89** |
| PCK1 | phosphoenolpyruvate carboxykinase 1 | **5.365** | **3.35E-46** |
| CHI3L1 | chitinase 3 like 1 | **5.322** | **6.19E-73** |
| NKX1-2 | NK1 homeobox 2 | **5.277** | **1.86E-35** |
| ASS1 | argininosuccinate synthase 1 | **5.233** | **2.47E-276** |
| AGXT | alanine--glyoxylate and serine--pyruvate aminotransferase | **5.232** | **9.35E-49** |
| CPN2 | carboxypeptidase N subunit 2 | **5.068** | **5.87E-107** |
| KCNQ1 | potassium voltage-gated channel subfamily Q member 1 | **5.004** | **2.73E-37** |
| LIPC | lipase C, hepatic type | **4.959** | **3.63E-229** |
| TKTL1 | transketolase like 1 | **4.928** | **4.59E-28** |
| HAL | histidine ammonia-lyase | **4.909** | **1.82E-54** |
| ITIH3 | inter-alpha-trypsin inhibitor heavy chain 3 | **4.870** | **7.87E-30** |
| STAR | steroidogenic acute regulatory protein | **4.826** | **8.18E-163** |
| SLC9A3 | solute carrier family 9 member A3 | **4.817** | **4.51E-18** |
| ADH4 | alcohol dehydrogenase 4 (class II), pi polypeptide | **4.808** | **5.58E-18** |
| CLNK | cytokine dependent hematopoietic cell linker | **4.767** | **1.19E-17** |
| APOC3 | apolipoprotein C3 | **4.748** | **4.58E-229** |
| RBP2 | retinol binding protein 2 | **4.720** | **6.88E-75** |
| SLC22A6 | solute carrier family 22 member 6 | **4.711** | **1.47E-58** |
| INMT | indolethylamine N-methyltransferase | **4.699** | **3.91E-149** |
| GOLGA7B | golgin A7 family member B | **4.608** | **1.15E-48** |
| ELN | elastin | **4.605** | **2.83E-69** |
| C1QL2 | complement C1q like 2 | **4.579** | **1.20E-23** |
| APOA5 | apolipoprotein A5 | **4.572** | **2.25E-27** |
| LGR6 | leucine rich repeat containing G protein-coupled receptor 6 | **4.559** | **4.29E-132** |
| MYH6 | myosin heavy chain 6 | **4.558** | **4.30E-175** |
| MT1E | metallothionein 1E | **4.545** | **7.43E-41** |
| GCGR | glucagon receptor | **4.487** | **3.73E-131** |
| ABLIM3 | actin binding LIM protein family member 3 | **4.485** | **6.06E-36** |
| SERPINA5 | serpin family A member 5 | **4.454** | **9.23E-173** |
| GALNT18 | polypeptide N-acetylgalactosaminyltransferase 18 | **4.442** | **2.39E-76** |
| SLC2A7 | solute carrier family 2 member 7 | **4.370** | **1.23E-45** |
| GABBR2 | gamma-aminobutyric acid type B receptor subunit 2 | **4.350** | **2.05E-27** |
| GRIK3 | glutamate ionotropic receptor kainate type subunit 3 | **4.348** | **5.49E-19** |
| SLC10A1 | solute carrier family 10 member 1 | **4.299** | **2.09E-35** |
| RHBG | Rh family B glycoprotein | **4.297** | **9.05E-54** |
| CACNA1G | calcium voltage-gated channel subunit alpha1 G | **4.291** | **4.39E-13** |
| COL22A1 | collagen type XXII alpha 1 chain | **4.245** | **1.78E-143** |
| MT1F | metallothionein 1F | **4.224** | **1.93E-24** |
| SIM1 | SIM bHLH transcription factor 1 | **4.189** | **1.67E-15** |
| TRIM50 | tripartite motif containing 50 | **4.156** | **1.16E-17** |
| MYBPC3 | myosin binding protein C3 | **4.136** | **5.42E-58** |
| MT2A | metallothionein 2A | **4.131** | **1.54E-46** |
| DNASE1L3 | deoxyribonuclease 1 like 3 | **4.092** | **3.45E-13** |
| ORM1 | orosomucoid 1 | **4.067** | **8.69E-55** |
| NMUR1 | neuromedin U receptor 1 | **4.056** | **8.37E-74** |
| SORCS2 | sortilin related VPS10 domain containing receptor 2 | **4.055** | **5.62E-51** |
| HR | HR lysine demethylase and nuclear receptor corepressor | **4.034** | **9.50E-19** |
| UTS2R | urotensin 2 receptor | **4.032** | **1.09E-38** |
| TBX2 | T-box transcription factor 2 | **4.027** | **7.58E-78** |
| PHF24 | PHD finger protein 24 | **4.022** | **3.31E-53** |
| SLC6A12 | solute carrier family 6 member 12 | **4.008** | **2.01E-47** |
| AHNAK2 | AHNAK nucleoprotein 2 | **3.990** | **9.66E-101** |
| NKX2-8 | NK2 homeobox 8 | **3.982** | **6.57E-14** |
| PRODH2 | proline dehydrogenase 2 | **3.979** | **2.88E-92** |
| HAO2 | hydroxyacid oxidase 2 | **3.972** | **9.90E-24** |
| REN | renin | **3.970** | **1.02E-24** |
| ETNPPL | ethanolamine-phosphate phospho-lyase | **3.951** | **5.31E-18** |
| LRTM2 | leucine rich repeats and transmembrane domains 2 | **3.915** | **2.67E-68** |
| PHLDA2 | pleckstrin homology like domain family A member 2 | **3.910** | **1.01E-22** |
| SLC22A7 | solute carrier family 22 member 7 | **3.883** | **3.67E-31** |
| LRRC15 | leucine rich repeat containing 15 | **3.875** | **1.06E-15** |
| PGLYRP2 | peptidoglycan recognition protein 2 | **3.852** | **6.60E-17** |
| TBX1 | T-box transcription factor 1 | **3.836** | **1.61E-34** |
| BRINP1 | BMP/retinoic acid inducible neural specific 1 | **3.826** | **1.55E-41** |
| NEFM | neurofilament medium | **3.813** | **4.54E-12** |
| NEFL | neurofilament light | **3.795** | **2.45E-24** |
| COTL1 | coactosin like F-actin binding protein 1 | **3.783** | **5.59E-234** |
| SUSD3 | sushi domain containing | **3.757** | **6.65E-73** |
| MLXIPL | MLX interacting protein like | **3.742** | **1.57E-84** |
| SLC51A | solute carrier family 51 subunit alpha | **3.731** | **7.85E-67** |
| CHRNA4 | cholinergic receptor nicotinic alpha 4 subunit | **3.727** | **5.29E-15** |
| C3 | complement C3 | **3.726** | **9.65E-46** |
| PCP4L1 | Purkinje cell protein 4 like 1 | **3.719** | **1.72E-48** |
| SECTM1 | secreted and transmembrane 1 | **3.697** | **1.02E-26** |
| SLC6A3 | solute carrier family 6 member 3 | **3.675** | **1.15E-12** |
| SH3RF3 | SH3 domain containing ring finger 3 | **3.667** | **7.13E-18** |
| G6PC | glucose-6-phosphatase catalytic subunit | **3.667** | **9.98E-11** |
| ITIH1 | inter-alpha-trypsin inhibitor heavy chain 1 | **3.640** | **3.13E-78** |
| GLTPD2 | glycolipid transfer protein domain containing 2 | **3.640** | **1.87E-51** |
| GRIA1 | glutamate ionotropic receptor AMPA type subunit 1 | **3.633** | **3.26E-12** |
| ATP2B2 | ATPase plasma membrane Ca2+ transporting 2 | **3.591** | **8.98E-35** |
| HCAR3 | hydroxycarboxylic acid receptor 3 | **3.588** | **1.68E-22** |
| KCNF1 | potassium voltage-gated channel modifier subfamily F member 1 | **3.530** | **8.94E-50** |
| RASD1 | ras related dexamethasone induced 1 | **3.529** | **2.03E-103** |
| SPIB | Spi-B transcription factor | **3.512** | **7.42E-11** |
| SLC18A1 | solute carrier family 18 member A1 | **3.509** | **1.07E-24** |
| CEACAM1 | CEA cell adhesion molecule 1 | **3.508** | **4.89E-19** |
| SULT1A1 | sulfotransferase family 1A member 1 | **3.506** | **4.57E-108** |
| TAC4 | tachykinin precursor 4 | **3.505** | **2.20E-12** |
