# Supplementary material for: Loss of FOXA2 induces ER stress and hepatic steatosis and alters developmental gene expression in human iPSC-derived hepatocytes
Source: Cell Death Dis. 2022 Aug 16;13(8):713. doi: 10.1038/s41419-022-05158-0 (PMC9381545; doi:10.1038/s41419-022-05158-0)
Supplement: Supplementary file 11 — Supplementary Table 5 [file 41419_2022_5158_MOESM11_ESM.docx]

**Supplementary Table 5. Top downregulated genes in hepatic progenitors derived from FOXA2^-/-^ iPSCs compared with WT controls**

| **Gene name** | **Gene description** | **Log2 FC** | ***P-*value** |
| --- | --- | --- | --- |
| PEG3 | paternally expressed 3 | -7.804 | 3.19E-69 |
| ZNF736 | zinc finger protein 736 | -6.575 | 1.15E-50 |
| ZNF208 | zinc finger protein 20 | -5.935 | 2.47E-76 |
| ZNF676 | zinc finger protein 676 | -5.922 | 3.31E-41 |
| ZNF559 | zinc finger protein 559 | -5.546 | 3.11E-39 |
| ZNF667 | zinc finger protein 667 | -5.500 | 5.16E-36 |
| UCN3 | urocortin 3 | -5.301 | 7.78E-27 |
| SLFN13 | schlafen family member 13 | -5.263 | 7.06E-82 |
| ZNF471 | zinc finger protein 471 | -5.172 | 1.99E-32 |
| ZNF257 | zinc finger protein 257 | -4.906 | 1.75E-20 |
| UNC5D | unc-5 netrin receptor D | -4.851 | 8.41E-116 |
| GABRP | gamma-aminobutyric acid type A receptor subunit pi | -4.696 | 3.53E-76 |
| DIRAS3 | DIRAS family GTPase 3 | -4.659 | 5.48E-40 |
| RGS13 | regulator of G protein signaling 13 | -4.592 | 4.61E-21 |
| FGF14 | fibroblast growth factor 14 | -4.510 | 2.35E-45 |
| ZNF726 | zinc finger protein 726 | -4.492 | 7.36E-19 |
| RGPD2 | RANBP2 like and GRIP domain containing 2 | -4.485 | 8.76E-19 |
| SCN2A | sodium voltage-gated channel alpha subunit 2 | -4.394 | 2.19E-100 |
| TMEM132C | transmembrane protein 132C | -4.335 | 2.85E-30 |
| KCNV1 | potassium voltage-gated channel modifier subfamily V member 1 | -4.291 | 6.70E-23 |
| ONECUT3 | one cut homeobox 3 | -4.270 | 1.82E-14 |
| PRRX1 | paired related homeobox 1 | -4.126 | 1.14E-12 |
| TYRP1 | tyrosinase related protein 1 | -4.061 | 1.95E-36 |
| BICDL2 | BICD family like cargo adaptor 2 | -4.021 | 2.31E-23 |
| COL6A3 | collagen type VI alpha 3 chain | -4.011 | 2.94E-138 |
| VSIG1 | V-set and immunoglobulin domain containing 1 | -4.003 | 4.21E-34 |
| FOLH1 | folate hydrolase 1 | -3.890 | 3.78E-13 |
| NLRP2 | NLR family pyrin domain containing 2 | -3.825 | 3.67E-39 |
| ZNF835 | zinc finger protein 835 | -3.731 | 1.02E-10 |
| VAT1L | vesicle amine transport 1 like] | -3.677 | 2.16E-52 |
| PLAT | plasminogen activator, tissue type | -3.645 | 2.82E-38 |
| CHCHD2 | coiled-coil-helix-coiled-coil-helix domain containing 2 | -3.627 | 4.23E-16 |
| KCND2 | potassium voltage-gated channel subfamily D member 2 | -3.618 | 3.31E-15 |
| PCDHA6 | protocadherin alpha 6 | -3.612 | 3.58E-20 |
| SYNPR | synaptoporin | -3.597 | 3.40E-12 |
| TNNT1 | troponin T1, slow skeletal type | -3.541 | 1.72E-14 |
| HAND1 | heart and neural crest derivatives expressed 1 | -3.539 | 2.11E-71 |
| OTX2 | orthodenticle homeobox 2 | -3.510 | 1.44E-39 |
| ISL1 | ISL LIM homeobox 1 | -3.480 | 2.94E-11 |
| AARD | alanine and arginine rich domain containing protein | -3.477 | 3.28E-40 |
| PNPO | pyridoxamine 5'-phosphate oxidase | -3.467 | 8.15E-15 |
| NPY5R | neuropeptide Y receptor Y5 | -3.443 | 2.01E-11 |
| PRRT1B | proline rich transmembrane protein 1B | -3.438 | 3.24E-22 |
| MMP25 | matrix metallopeptidase 25 | -3.419 | 9.03E-12 |
| SPOCK3 | SPARC (osteonectin), cwcv and kazal like domains proteoglycan 3 | -3.395 | 2.28E-14 |
| ONECUT2 | one cut homeobox 2 | -3.377 | 3.06E-129 |
| MAB21L2 | mab-21 like 2 | -3.364 | 3.78E-24 |
| GSTM5 | glutathione S-transferase mu 5 | -3.354 | 6.38E-13 |
| ACP3 | acid phosphatase 3 | -3.329 | 1.05E-18 |
| MMP10 | matrix metallopeptidase 10 | -3.328 | 3.52E-10 |
| ADCY2 | adenylate cyclase 2 | -3.308 | 2.23E-58 |
| SLITRK6 | SLIT and NTRK like family member 6 | -3.266 | 1.68E-08 |
| PCDH15 | protocadherin related 15 | -3.248 | 3.79E-85 |
| MISP | mitotic spindle positioning | -3.246 | 1.62E-19 |
| LOXL3 | lysyl oxidase like 3 | -3.245 | 1.33E-31 |
| CHL1 | cell adhesion molecule L1 like | -3.234 | 6.55E-44 |
| RIMBP2 | RIMS binding protein 2 | -3.225 | 6.48E-33 |
| VTCN1 | V-set domain containing T cell activation inhibitor 1 | -3.216 | 3.39E-21 |
| DGKB | diacylglycerol kinase beta | -3.154 | 6.12E-08 |
| IFITM1 | interferon induced transmembrane protein 1 | -3.147 | 1.32E-08 |
| KCNT2 | potassium sodium-activated channel subfamily T member 2 | -3.143 | 2.97E-17 |
| PLEKHG4B | pleckstrin homology and RhoGEF domain containing G4B | -3.140 | 4.84E-64 |
| FAM181B | family with sequence similarity 181 member B | -3.117 | 8.64E-13 |
| SERPINB13 | serpin family B member 13 | -3.112 | 2.24E-31 |
| GLP1R | glucagon like peptide 1 receptor | -3.092 | 6.11E-71 |
| FOXF1 | forkhead box F1 | -3.089 | 6.64E-12 |
| DNAH8 | dynein axonemal heavy chain 8 | -3.065 | 1.23E-52 |
| CADPS | calcium dependent secretion activator | -3.060 | 2.30E-69 |
| TOX | thymocyte selection associated high mobility group box | -3.053 | 2.28E-43 |
| XIRP1 | xin actin binding repeat containing 1 | -3.022 | 7.69E-08 |
| COL21A1 | collagen type XXI alpha 1 chain | -3.005 | 2.77E-48 |
| FSD1 | fibronectin type III and SPRY domain containing 1 | -2.994 | 3.55E-20 |
| FIBIN | fin bud initiation factor homolog | -2.969 | 3.28E-07 |
| PRRT4 | proline rich transmembrane protein 4 | -2.960 | 1.36E-10 |
| ART4 | ADP-ribosyltransferase 4 (inactive) (Dombrock blood group) | -2.957 | 1.07E-06 |
| LGI4 | leucine rich repeat LGI family member 4 | -2.956 | 8.48E-37 |
| SLIT1 | slit guidance ligand 1 | -2.945 | 1.52E-26 |
| KRT23 | keratin 23 [Source:HGNC Symbol;Acc:HGNC:6438] | -2.927 | 4.99E-15 |
| CCN4 | cellular communication network factor 4 | -2.908 | 1.23E-12 |
| SLC4A4 | solute carrier family 4 member 4 | -2.901 | 1.85E-25 |
| TMEM158 | transmembrane protein 158 | -2.898 | 6.11E-68 |
| ERBB4 | erb-b2 receptor tyrosine kinase 4 | -2.898 | 3.50E-31 |
| ATP8B3 | ATPase phospholipid transporting 8B3 | -2.889 | 6.46E-25 |
| PCDHGA10 | protocadherin gamma subfamily A, 10 | -2.887 | 7.84E-23 |
| SEMA3F | semaphorin 3F | -2.881 | 4.64E-20 |
| CAMK1G | calcium/calmodulin dependent protein kinase IG | -2.880 | 1.96E-07 |
| TSHZ3 | teashirt zinc finger homeobox 3 | -2.867 | 2.15E-22 |
| ZXDA | zinc finger X-linked duplicated A | -2.863 | 4.12E-10 |
| PALMD | palmdelphin | -2.859 | 3.85E-24 |
| FXYD6 | FXYD domain containing ion transport regulator 6 | -2.856 | 1.46E-67 |
| GRIN2A | glutamate ionotropic receptor NMDA type subunit 2A | -2.854 | 8.63E-36 |
| NUP62CL | nucleoporin 62 C-terminal like | -2.838 | 6.24E-11 |
| CAMK1D | calcium/calmodulin dependent protein kinase ID | -2.830 | 5.17E-14 |
| SAPCD2 | suppressor APC domain containing 2 | -2.818 | 3.48E-40 |
| SLC6A16 | solute carrier family 6 member 16 | -2.814 | 3.49E-16 |
| DCN | decorin | -2.805 | 3.03E-08 |
| ST8SIA3 | ST8 alpha-N-acetyl-neuraminide alpha-2,8-sialyltransferase 3 | -2.803 | 1.31E-14 |
| ADAM33 | ADAM metallopeptidase domain 33 | -2.798 | 2.82E-09 |
| HHEX | hematopoietically expressed homeobox | -2.788 | 4.40E-60 |
| SVEP1 | sushi, von Willebrand factor type A, EGF and pentraxin domain containing 1 | -2.786 | 1.95E-19 |
| WFDC2 | WAP four-disulfide core domain 2 | -2.778 | 3.64E-33 |
| TSHZ2 | teashirt zinc finger homeobox 2 | -2.775 | 2.07E-11 |
| GRHL2 | grainyhead like transcription factor 2 | -2.770 | 7.86E-36 |
| PDX1 | pancreatic and duodenal homeobox 1 | -2.760 | 8.85E-34 |
| SDK1 | sidekick cell adhesion molecule 1 | -2.737 | 6.18E-44 |
| ZNF469 | zinc finger protein 469 | -2.735 | 5.15E-13 |
| NDNF | neuron derived neurotrophic factor | -2.732 | 9.13E-19 |
| CDH3 | cadherin 3 | -2.710 | 6.41E-62 |
| TGFBI | transforming growth factor beta induced | -2.701 | 4.70E-49 |
| ZNF572 | zinc finger protein 572 | -2.699 | 4.50E-06 |
| DCX | doublecortin | -2.689 | 2.61E-16 |
| ADGRG2 | adhesion G protein-coupled receptor G2 | -2.688 | 5.09E-102 |
| APCDD1 | APC down-regulated 1 | -2.685 | 2.83E-23 |
| HAS2 | hyaluronan synthase 2 | -2.684 | 7.88E-29 |
| EPHA7 | EPH receptor A7 | -2.677 | 1.82E-99 |
| NOL4 | nucleolar protein 4 | -2.672 | 6.72E-55 |
| TFAP2B | transcription factor AP-2 beta | -2.669 | 9.66E-06 |
| CHST6 | carbohydrate sulfotransferase 6 | -2.667 | 6.04E-28 |
| COL3A1 | collagen type III alpha 1 chain | -2.667 | 2.91E-107 |
| GRIK5 | glutamate ionotropic receptor kainate type subunit 5 | -2.661 | 3.56E-10 |
| PSG8 | pregnancy specific beta-1-glycoprotein 8 | -2.655 | 4.49E-09 |
| ZNF728 | zinc finger protein 728 | -2.637 | 1.88E-05 |
| VWA5B2 | von Willebrand factor A domain containing 5B2 | -2.634 | 1.46E-07 |
| PLN | phospholamban | -2.632 | 2.04E-13 |
| KCNMA1 | potassium calcium-activated channel subfamily M alpha 1 | -2.6308 | 2.58E-12 |
| LRRC75A | leucine rich repeat containing 75A | -2.628 | 1.06E-09 |
| SMYD1 | SET and MYND domain containing 1 | -2.618 | 2.10E-05 |
| DPP6 | dipeptidyl peptidase like 6 | -2.607 | 3.04E-49 |
| TNNI1 | troponin I1, slow skeletal type | -2.599 | 3.56E-14 |
| FGF19 | fibroblast growth factor 19 | -2.598 | 1.17E-05 |
| ZNF98 | zinc finger protein 98 | -2.589 | 2.64E-05 |
| BSPRY | B-box and SPRY domain containing | -2.585 | 8.64E-20 |
| TAFA2 | TAFA chemokine like family member 2 | -2.571 | 1.44E-10 |
| SCN9A | sodium voltage-gated channel alpha subunit 9 | -2.571 | 4.52E-57 |
| IGFBP5 | insulin like growth factor binding protein 5 | -2.570 | 1.19E-59 |
| CD300A | CD300a molecule | -2.556 | 1.45E-14 |
| ALX4 | ALX homeobox 4 | -2.548 | 2.01E-05 |
| NT5E | 5'-nucleotidase ecto | -2.541 | 8.39E-18 |
| LIX1 | limb and CNS expressed 1 | -2.538 | 2.99E-14 |
| HAND2 | heart and neural crest derivatives expressed 2 | -2.536 | 1.51E-14 |
| SYK | spleen associated tyrosine kinase | -2.535 | 1.28E-11 |
| TRPC3 | transient receptor potential cation channel subfamily C member 3 | -2.535 | 1.01E-05 |
| SMAD9 | SMAD family member 9 | -2.534 | 1.13E-26 |
| CNTN3 | contactin 3 | -2.528 | 2.40E-28 |
| PRR36 | proline rich 36 | -2.522 | 3.65E-26 |
| HLA-DOA | major histocompatibility complex, class II, DO alpha | -2.517 | 6.07E-10 |
| MS4A8 | membrane spanning 4-domains A8 | -2.514 | 1.96E-07 |
| TBX20 | T-box transcription factor 20 | -2.505 | 2.35E-09 |
| PCLO | piccolo presynaptic cytomatrix protein | -2.504 | 1.46E-46 |
| CENPF | centromere protein F | -2.504 | 1.64E-88 |
| KCNC3 | potassium voltage-gated channel subfamily C member 3 | -2.503 | 2.80E-14 |
| RGS1 | regulator of G protein signaling 1 | -2.502 | 5.21E-05 |
| LTBP4 | latent transforming growth factor beta binding protein 4 | -2.500 | 1.99E-21 |
