# Supplementary material for: Loss of FOXA2 induces ER stress and hepatic steatosis and alters developmental gene expression in human iPSC-derived hepatocytes
Source: Cell Death Dis. 2022 Aug 16;13(8):713. doi: 10.1038/s41419-022-05158-0 (PMC9381545; doi:10.1038/s41419-022-05158-0)
Supplement: Supplementary file 12 — Supplementary Table 6 [file 41419_2022_5158_MOESM12_ESM.docx]

**Supplementary Table 6. Top upregulated genes in mature hepatocytes derived from FOXA2^-/-^ iPSCs compared with WT controls**

| **Gene name** | **Gene description** | **log2_fc** | ***p-*value** |
| --- | --- | --- | --- |
| C7 | Complement C7 | **5.202** | 7.48461E-74 |
| DLK1 | Delta like non-canonical Notch ligand 1 | **4.001** | 6.61667E-75 |
| MFAP4 | Microfibril associated protein 4 | **3.800** | 4.51748E-48 |
| REN | Renin | **3.503** | 4.40573E-22 |
| AKR1B15 | Aldo-keto reductase family 1 member B15 | **3.495** | 1.13355E-22 |
| ITGA8 | Integrin subunit alpha 8 | **3.458** | 8.41477E-24 |
| DES | Desmin | **3.378** | 4.00278E-26 |
| MMP2 | Matrix metallopeptidase 2 | **3.345** | 2.74509E-43 |
| COL1A2 | Collagen type I alpha 2 chain | **3.342** | 4.89234E-89 |
| TFPI2 | Tissue factor pathway inhibitor 2 | **3.233** | 8.85819E-43 |
| COL8A2 | Collagen type VIII alpha 2 chain | **3.159** | 1.4137E-38 |
| COLEC11 | Collectin subfamily member 11 | **3.100** | 3.30107E-18 |
| CFH | Complement factor H | **3.059** | 7.94316E-24 |
| GGT5 | Gamma-glutamyltransferase 5 | **3.049** | 1.11515E-16 |
| PAPPA | Pappalysin 1 | **2.962** | 5.41265E-51 |
| COL15A1 | Collagen type XV alpha 1 chain | **2.940** | 2.49451E-22 |
| CD248 | CD248 molecule | **2.935** | 1.79015E-15 |
| ISM2 | Isthmin 2 | **2.926** | 1.84124E-59 |
| LAMC3 | Laminin subunit gamma 3 | **2.908** | 3.75748E-15 |
| AQP1 | Aquaporin 1 | **2.895** | 7.12982E-18 |
| PENK | Proenkephalin | **2.859** | 3.21483E-19 |
| COL3A1 | Collagen type III alpha 1 chain | **2.857** | 8.02771E-55 |
| FABP2 | Fatty acid binding protein 2 | **2.838** | 1.15848E-30 |
| BGN | Biglycan | **2.838** | 3.21097E-15 |
| GBP4 | Guanylate binding protein 4 | **2.833** | 3.09117E-15 |
| DSC3 | Desmocollin 3 | **2.821** | 2.11181E-25 |
| FBLN2 | Fibulin 2 | **2.805** | 8.52118E-45 |
| C11orf86 | Chromosome 11 open reading frame 86 | **2.797** | 1.13077E-15 |
| COL6A3 | Collagen type VI alpha 3 chain | **2.785** | 1.84385E-22 |
| LRRC32 | Leucine rich repeat containing 32 | **2.783** | 3.39492E-14 |
| PNMT | Phenylethanolamine N-methyltransferase | **2.773** | 3.22157E-31 |
| CAVIN2 | Caveolae associated protein 2 | **2.773** | 1.25004E-15 |
| TBX18 | T-box transcription factor 18 | **2.772** | 1.71289E-17 |
| HAPLN1 | Hyaluronan and proteoglycan link protein 1 | **2.702** | 2.05784E-15 |
| CDH11 | Cadherin 11 | **2.682** | 1.72071E-29 |
| RBP2 | Retinol binding protein 2 | **2.678** | 2.99409E-31 |
| SLC26A3 | Solute carrier family 26 member 3 | **2.672** | 2.74251E-23 |
| GNG2 | G protein subunit gamma 2 | **2.662** | 4.54882E-15 |
| WT1 | WT1 transcription factor | **2.630** | 8.81929E-13 |
| ITGA4 | Integrin subunit alpha 4 | **2.629** | 1.88461E-12 |
| FREM1 | FRAS1 related extracellular matrix 1 | **2.628** | 3.91189E-22 |
| BMP5 | Bone morphogenetic protein 5 | **2.624** | 1.03193E-14 |
| SFRP5 | Secreted frizzled related protein 5 | **2.617** | 4.84838E-23 |
| SFRP1 | Secreted frizzled related protein 1 | **2.592** | 4.68026E-39 |
| SLC13A4 | Solute carrier family 13 member 4 | **2.586** | 3.31823E-16 |
| GNB3 | G protein subunit beta 3 | **2.581** | 1.54539E-14 |
| COL1A1 | Collagen type I alpha 1 chain | **2.562** | 3.35568E-52 |
| POSTN | Periostin | **2.552** | 9.10892E-32 |
| KCNQ5 | Potassium voltage-gated channel subfamily Q member 5 | **2.545** | 7.93459E-19 |
