# Supplementary material for: Loss of FOXA2 induces ER stress and hepatic steatosis and alters developmental gene expression in human iPSC-derived hepatocytes
Source: Cell Death Dis. 2022 Aug 16;13(8):713. doi: 10.1038/s41419-022-05158-0 (PMC9381545; doi:10.1038/s41419-022-05158-0)
Supplement: Supplementary file 13 — Supplementary Table 7 [file 41419_2022_5158_MOESM13_ESM.docx]

**Supplementary Table 7. Top downregulated genes in mature hepatocytes derived from FOXA2^-/-^ iPSCs compared with WT controls**

| **Gene ID** | **Gene ID** | **Log1 FC** | ***P-*value** |
| --- | --- | --- | --- |
| MLPH | Melanophilin | **-3.073** | 1.29890715703393e-34 |
| GADL1 | Glutamate decarboxylase like 1 | **-2.59** | 7.48882924992621e-39 |
| DNER | Delta/notch like EGF repeat containing | **-2.58** | 7.52900487741861e-41 |
| SPTB | Spectrin beta, erythrocytic | **-2.51** | 1.66712909305802e-30 |
| SLC5A5 | solute carrier family 5 member 5 | **-2.498** | 1.05226527518809e-31 |
| GABRA4 | Gamma-aminobutyric acid type A receptor subunit alpha4 | **-2.49** | 1.39673448047801e-25 |
| SPOCK2 | SPARC (osteonectin), cwcv and kazal like domains proteoglycan 2 | **-2.46** | 1.05925727765333e-65 |
| CTSE | Cathepsin E | **-2.404** | 1.33439461753965e-17 |
| AKR1C2 | Aldo-keto reductase family 1 member C2 | **-2.355** | 1.34793333846422e-25 |
| NR2E3 | Nuclear receptor subfamily 2 group E member 3 | **-2.313** | 4.99147182853023e-13 |
| HOXA1 | Homeobox A1 | **-2.306** | 8.45454429286098e-12 |
| DNAH8 | Dynein axonemal heavy chain 8 | **-2.274** | 1.17421231052808e-10 |
| RAB27B | RAB27B, member RAS oncogene family | **-2.239** | 1.82946024754521e-51 |
| CDH7 | Cadherin 7 | **-2.215** | 2.6628399197478e-17 |
| HOPX | HOP homeobox | **-2.076** | 1.09721192844155e-09 |
| PPEF1 | Protein phosphatase with EF-hand domain 1 | **-2.064** | 1.00977150119204e-12 |
| SLC10A4 | Solute carrier family 10 member 4 | **-2.060** | 7.08368398531283e-16 |
| VWA2 | Von Willebrand factor A domain containing 2 | **-2.053** | 4.55851879400376e-19 |
| PRIMA1 | Proline rich membrane anchor 1 | **-2.034** | 1.10502433092436e-07 |
| WARS1 | Tryptophanyl-tRNA synthetase 1 | **-1.943** | 4.08728920967767e-40 |
| HEPACAM2 | HEPACAM family member 2 | **-1.940** | 4.25902867280724e-13 |
| GPR78 | G protein-coupled receptor 78 | **-1.940** | 6.43691799930229e-08 |
| GLP1R | Glucagon like peptide 1 receptor | **-1.900** | 2.38923944584904e-13 |
| MFSD6L | Major facilitator superfamily domain containing 6 like | **-1.898** | 9.57998808306636e-16 |
| GPX2 | Glutathione peroxidase 2 | **-1.879** | 7.00478738167992e-07 |
| FAR2 | Fatty acyl-CoA reductase 2 | **-1.856** | 4.91329443712082e-12 |
| KRT23 | Keratin 23 | **-1.814** | 2.29172728035771e-06 |
| SLC16A12 | Solute carrier family 16 member 12 | **-1.804** | 1.35774130201781e-11 |
| CYP1A1 | Cytochrome P450 family 1 subfamily A member 1 | **-1.794** | 4.83410104189643e-29 |
| PCAT14 | Prostate cancer associated transcript 14 | **-1.792** | 4.55856579650388e-22 |
| PRSS1 | Serine protease 1 | **-1.778** | 3.1491976471961e-09 |
| PTPRT | Protein tyrosine phosphatase receptor type T | **-1.774** | 4.13129679481878e-14 |
| ADAMTS16 | ADAM metallopeptidase with thrombospondin type 1 motif 16 | **-1.770** | 6.89665541633874e-20 |
| PAPPA2 | Pappalysin 2 | **-1.767** | 1.577045558352e-07 |
| PSAPL1 | Prosaposin like 1 | **-1.728** | 4.5620330196932e-18 |
| CAMK2B | Calcium/calmodulin dependent protein kinase II beta | **-1.726** | 2.05274909573226e-07 |
| THSD4 | Thrombospondin type 1 domain containing 4 | **-1.724** | 1.21229942356165e-12 |
| FAM162B | Family with sequence similarity 162 member B | **-1.706** | 4.83152813559649e-06 |
| RIPOR2 | RHO family interacting cell polarization regulator 2 | **-1.690** | 9.0227936497739e-27 |
| HSD3BP4 | Hydroxy-delta-5-steroid dehydrogenase, 3 beta, pseudogene 4 | **-1.677** | 6.7548110445158e-13 |
| ANKRD33B | Ankyrin repeat domain 33B | **-1.676** | 6.93755362590708e-30 |
| CLDN18 | Claudin 18 | **-1.674** | 6.59804990609862e-11 |
| RFX6 | Regulatory factor X6 | **-1.638** | 1.95367154470846e-14 |
| SLC9A2 | Solute carrier family 9 member A2 | **-1.627** | 2.32913027555941e-06 |
| CPN1 | Carboxypeptidase N subunit 1 | **-1.606** | 2.98947729147335e-05 |
| BCO1 | Beta-carotene oxygenase 1 | **-1.599** | 6.92216635029569e-06 |
| CHAC1 | ChaC glutathione specific gamma-glutamylcyclotransferase 1 | **-1.598** | 4.479660293997e-17 |
| GSG1 | Germ cell associated 1 | **-1.593** | 2.82175847786675e-10 |
| ADCY1 | Adenylate cyclase 1 | **-1.579** | 6.39539119720193e-11 |
| FFAR4 | Free fatty acid receptor 4 | **-1.573** | 6.68469539128902e-07 |
| UGT2B15 | UDP glucuronosyltransferase family 2 member B15 | **-1.549** | 2.13068180387854e-11 |
| FRZB | Frizzled related protein | **-1.533** | 7.62837532461086e-21 |
| PSAT1 | Phosphoserine aminotransferase 1 | **-1.517** | 1.0240454694767e-24 |
| SLC26A7 | Solute carrier family 26 member 7 | **-1.515** | 1.35625472434426e-21 |
