# Supplementary material for: Loss of FOXA2 induces ER stress and hepatic steatosis and alters developmental gene expression in human iPSC-derived hepatocytes
Source: Cell Death Dis. 2022 Aug 16;13(8):713. doi: 10.1038/s41419-022-05158-0 (PMC9381545; doi:10.1038/s41419-022-05158-0)

Original Western blotting presented in **Figure 1C**

FOXA2

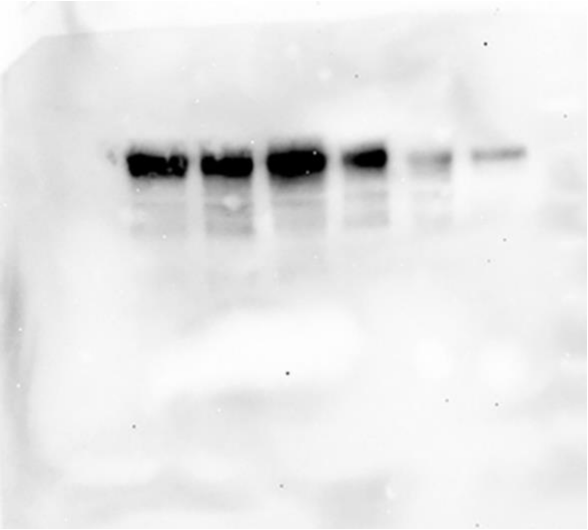

beta Actin

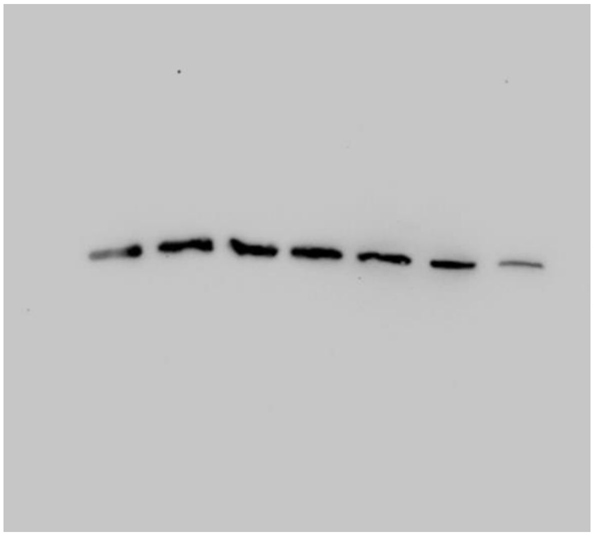

Original Western blotting presented in **Figure 1E**

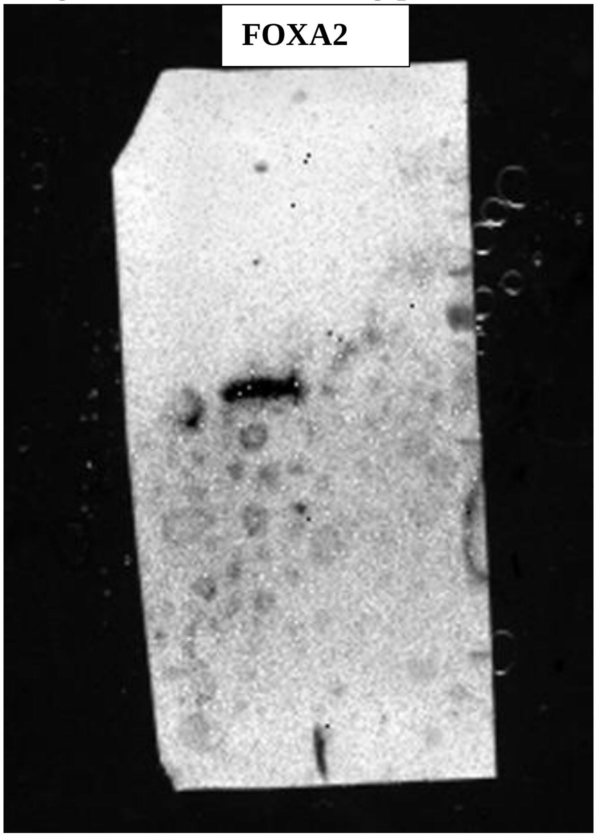

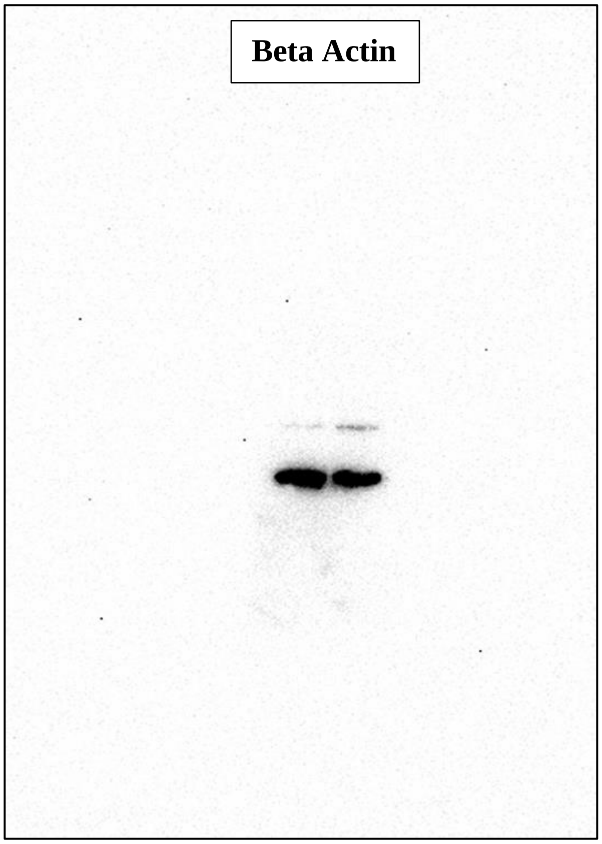

Original Western blotting presented in **Figure 2C**

AFP

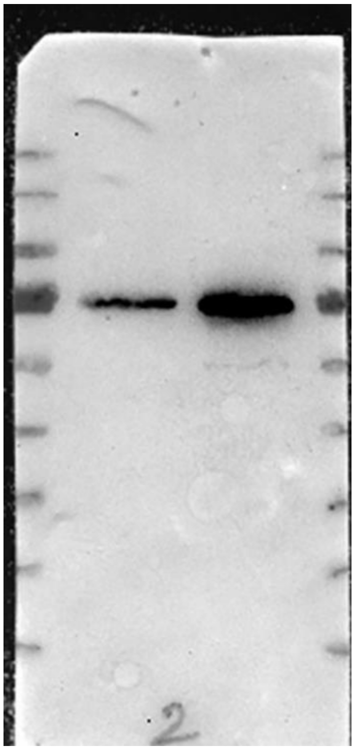

beta Actin

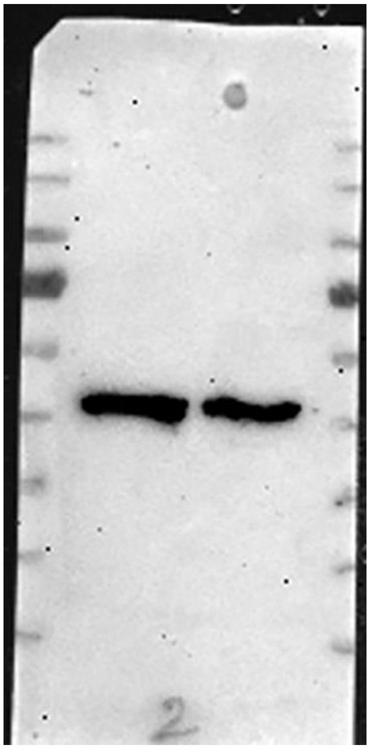

Original Western blotting presented in **Figure 2F**

ALBUMIN

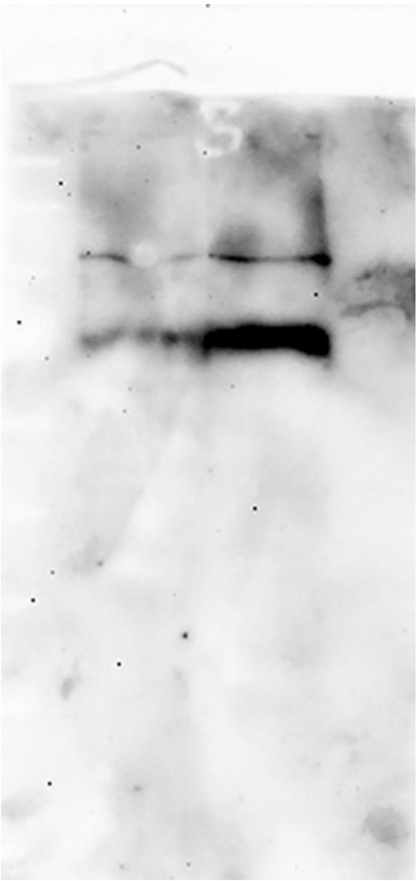

Beta Actin

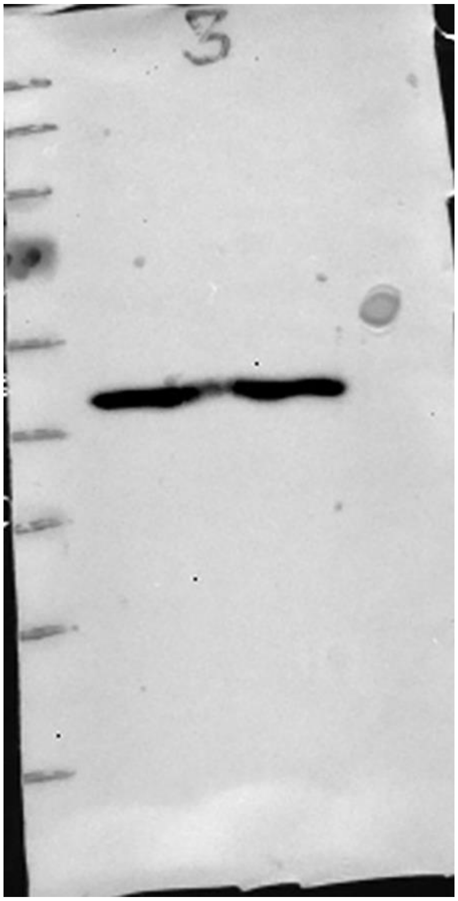

Original Western blotting presented in **Figure 6E**

Phospho AKT

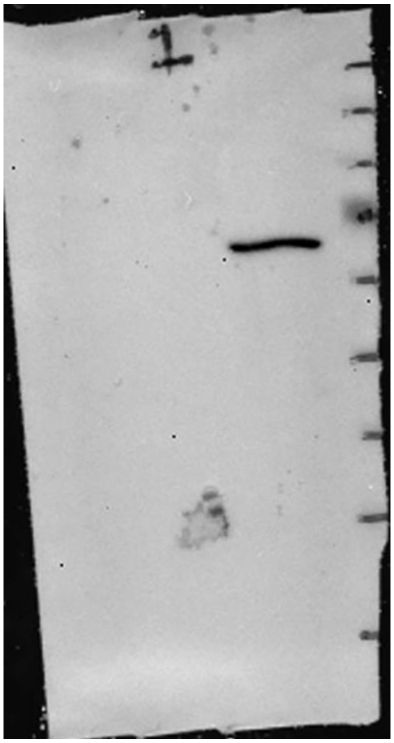

Total AKT

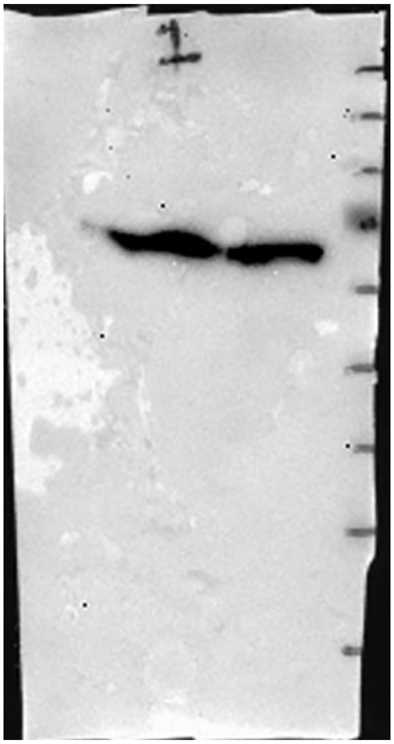

Original Western blotting presented in **Figure 7**

Phospho ERK

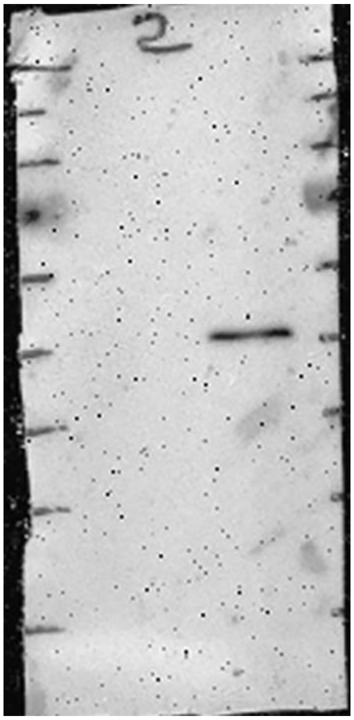

Total ERK

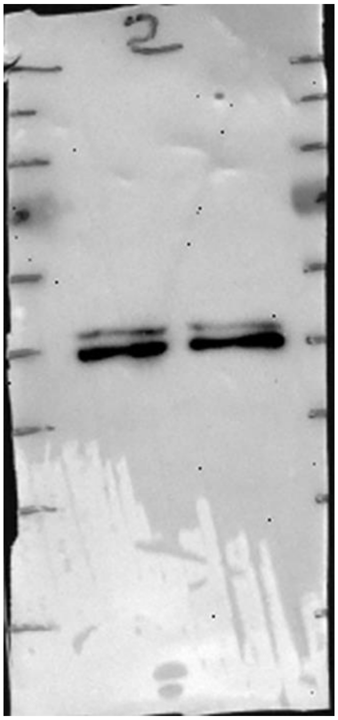

Beta Actin

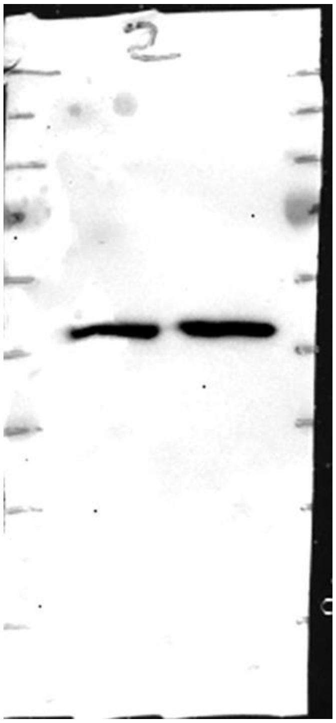

Supplement: Supplementary file 14 — Western Original Data File [file 41419_2022_5158_MOESM14_ESM.pdf]
